# Polyester-coated stainless-steel sheets using silica gel microparticles as surface pre-modifiers: a novel approach to determine selective serotonin reuptake inhibitors in saliva samples by direct infusion tandem mass spectrometry

**DOI:** 10.1007/s00604-025-07521-2

**Published:** 2025-09-09

**Authors:** Ana M. Pedraza-Soto, Rafael Lucena, Soledad Cárdenas

**Affiliations:** Affordable and Sustainable Sample Preparation (AS2P) Research Group, Departamento de Química Analítica, Instituto Químico para la Energía y el Medioambiente IQUEMA, Universidad de Córdoba, Campus Universitario de Rabanales, Edificio Marie Curie, E-14071 Córdoba, Spain

**Keywords:** Stainless-steel substrate, Silica gel microparticles, Adhesive tape, Polyester, Saliva analysis, SSRI

## Abstract

Stainless-steel substrates have grown in importance in the development of planar sorptive phases. However, the reduced wettability of polished sheets makes difficult their functionalization. This limitation can be solved by using amorphous silica gel microparticles as superficial guides. These particles increase both the wettability and the surface area of the final sorptive phases, which has a key influence on the extraction kinetics. In this article, double-sided thermal-resistant adhesive tape was used as the binder to pre-immobilize the silica particles over the stainless-steel surface, thus avoiding aggressive etching procedures. A linear polyester (synthesized by conventional free melt polycondensation from 1,4-butanediol and phenylsuccinic acid) was deposited onto the particulate area by the drop-casting technique. The resulting polyester-silica gel sheets (PSG-sheets) have been evaluated for the extraction of four selective serotonin reuptake inhibitors (SSRI) (fluoxetine, paroxetine, sertraline, and venlafaxine) in saliva samples, followed by direct infusion mass spectrometry analysis. The method allows the determination of the target compounds at the low µg·L^−1^ range, and it was successfully applied to analyze a saliva sample from a patient under paroxetine treatment. The method also fulfills the typical validation criteria in terms of precision (intrer-day precision better than 16.5% at the limit of quantification) and accuracy (relative recoveries in the interval 87–123% at the limit of quantification), both parameters having been evaluated at four concentration levels.

**Graphical Abstract:**

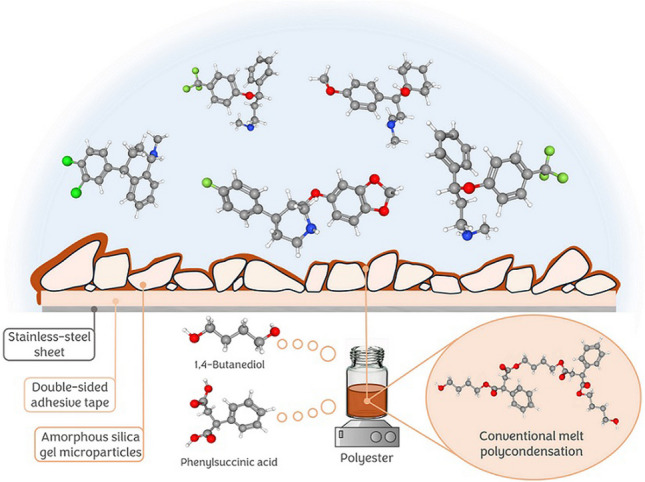

**Supplementary Information:**

The online version contains supplementary material available at 10.1007/s00604-025-07521-2.

## Introduction

Metallic substrates, such as stainless-steel, have gained significant importance for the preparation of thin-film microextraction (TFME) devices thanks to their intrinsic properties. Mechanical stability or electrical and thermal conductivity are considered key features to broaden the application field of the final materials, making possible the combination with specific sample processing devices, such as 96-well plate systems [1], or with other instrumental techniques, such as ambient ionization mass spectrometry (AIMS) in the so-called coated-blade spray technique (CBS) [2]. Moreover, these substrates can be tuned, allowing the selection of the sorbent material depending on the analytical problem. However, the smooth surface of metallic substrates offers reduced wettability, being a handicap for the application of simple modification approaches. In addition, the substrate presents a reduced surface area, making the immobilization of special sorptive phases (SP) needed to surpass this limitation.

The immobilization of porous sorptive polymers (PSP) is an interesting alternative to provide a high surface area [3]. They are synthesized in situ on the substrate by radical polymerization in the presence of a porogenic solvent to create homogeneous coatings with voids caused by the porogen elimination after the synthesis. In this way, *Azizi *et al*.* proposed the synthesis of a PSP onto stainless-steel sheets based on methacrylic acid (MMA) as monomer, ethylene glycol methacrylate (EGDMA) as crosslinker and octanol as porogen solvent [4].

Particulate sorptive materials (PSMs) are the most common way to provide active surface areas to stainless-steel sheets while ensuring good extraction capacity. PSM are mainly immobilized on the surface using polyacrylonitrile (PAN) as the polymeric binder, which encapsulates the particles and leaves them retained on the flat support. This preparation is easily achieved starting from a slurry where PSMs are dispersed in PAN solution, being subsequently deposited onto the sheet by simple techniques. In this sense, dip-coating technique has been reported to immobilize hydrophilic-lipophilic balance microparticles (HLB) [5], C18 [6], MWCNTs [7], metal–organic frameworks (MOFs) [8], and SiO_2_ nanoparticles [9]. In contrast, spray-coating technique has been proposed to deposit octadecyl-fibrous mesoporous silica nanospheres (C18-FMSNs) [10]. In all these approaches, prior to the phase deposition, the roughness of the metallic substrate must be increased by chemical (treating the surface with strong acids or bases) or mechanical etching (with sandpaper), thus compromising the operator’s safety.

Adhesive tapes are affordable and straightforward alternatives to immobilize PSM, minimizing solvent consumption and even avoiding the etching of the surface. By this approach, the immobilization of diatomaceous earth [11] and a biosorbent (Bract) [12] on stainless-steel by using double-sided adhesive tape as the binder has been reported. The procedure consists of simply introducing the glued area into the bulk particulate sorbent, which remains fully exposed on the surface of the stainless-steel to interact with the analytes. In combination with adhesive tapes, particulate materials can also be used as guiding substrates, permitting the direct deposition of polymers over the substrate by dip-coating. Recently, our research group developed this concept using amorphous silica gel microparticles as a guiding element for the deposition of commercial nylon-6 [13]. The microparticles were immobilized with double-sided adhesive tape on the stainless-steel sheets, improving the wettability of the sheets, which is essential for the dip-coating success.

Polyesters are synthetic biocompatible polymers widely used in medical applications, such as controlled drug release systems [14]. In addition, their intrinsic chemical resistance and thermal conductivity make them compatible with chemical and thermal elution strategies [15]. They have also been used as sorptive phase in microextraction thanks to their ability to interact with target analytes [16]. In this article, we evaluate the synthesis of polyester-coated metallic sheets by combining the guiding substrate concept with the drop-casting technique. For this purpose, amorphous silica gel microparticles were adhered to the stainless-steel sheets by means of thermal-resistant double-sided adhesive tapes. A linear polyester, synthesized by conventional melt polycondensation of phenylsuccinic acid and 1,4-butanediol, was subsequently deposited onto the particulate sheet, and cured in the oven at 235 ºC to evaporate the solvent. In this case, the diol acts as both monomer and synthesis medium, being a solvent-free polymerization method. The extraction efficiency was evaluated by determining four selective serotonin reuptake inhibitors (SSRI) (fluoxetine, paroxetine, sertraline, and venlafaxine) in saliva samples, and the sample preparation step was directly combined with mass spectrometry by direct infusion mass spectrometry analysis (DI-MS/MS). These analytes, considers as second-generation antidepressants, are currently the most prescribed medicaments for the treatment of depression and anxiety, among other conditions. For these reasons, their control in biosamples is of great importance to allow the therapeutic monitorization of these drugs. The developed approach was used to quantify a positive saliva sample from a volunteer undergoing paroxetine treatment, thus justifying the potential of the method to be used for this analytical problem.

## Experimental section

### Reagents and samples

Fluoxetine, paroxetine, sertraline, venlafaxine and their isotopically labeled compounds (fluoxetine-d_6_, paroxetine-d_6_, sertraline-d_3_ and venlafaxine-d_6_) were acquired from Sigma-Aldrich (Madrid, Spain). The preparation of the stock and working standards is detailed in electronic supporting material (ESM).

Stainless-steel sheets (304 austenitic, 0.1 mm thickness) were used as substrate for the preparation of polyester-silica gel sheets (PSG-sheets). The sheets were modified with thermal resistant double-sided adhesive tape (Wantouth, Amazon) and amorphous silica gel microparticles of different sizes (70–230 mesh, 28–200 mesh, and 35–70 mesh) purchased at Merck. Phenylsuccinic acid (purity > 99%, Fluka Chemica) and 1,4-butanediol (99%, Sigma-Aldrich) were selected as monomers for the polyester synthesis.

Blank oral fluids were obtained from healthy volunteers by passive drooling, making a pool to cover the inter-person variability. The pool was spiked with the target analytes and their internal standards (ISs), centrifuged 3 min at 11,068 g, and prior to the extraction procedure, the pH was set to 10 using ammonium hydroxide solution (30% v/v, Panreac, Barcelona, Spain). One saliva sample from a volunteer medically treated with paroxetine was collected, spiked with the ISs and pre-processed following the same procedure.

### Synthesis and characterization of the PSG-sheets

The polyester synthesis involves two stages. Firstly, a pre-polymer solution was synthesized by a catalyst-free melt polycondensation method [17]. For this purpose, phenylsuccinic acid (219 mg) was dissolved in 1,4-butanediol (1 mL) in a vial, maintaining the diacid:diol molar ratio at 1:10. The reaction takes place in the open-air atmosphere under continuous stirring (100 rpm) at 200 ºC keeping the vial in direct contact with a hot plate for 1 h. The temperature was controlled with a probe. A light brown viscous liquid is obtained as a pre-polymer, which is subsequently deposited on the stainless-steel substrate by adapting the procedure developed in previous work [13], which is detailed in Section 2 of ESM, to obtain the final polyester. PSG-sheets were not reused to avoid cross-contamination considering the simple and cost-effective fabrication procedure, since about 100 PSG-sheets can be prepared with a single batch of pre-polymer.

PSG-sheets were chemically characterized by attenuated total reflection infrared spectroscopy (ATR-IR) to monitor the reaction course using a Bruker Tensor 37 FT-IR spectrometer. In addition, the sheets were characterized by Scanning Electron Microscopy (SEM) and Energy-Dispersive X-ray spectroscopy (EDX). SEM and EDX measurements were developed at the Central Research Support Services (SCAI) of the University of Córdoba.

### Analytical procedure

The extraction workflow was carried out in HPLC vials, and the PSG-sheet was immobilized in a cap with pre-cut septa during the whole procedure, thus facilitating its handling. Initially, the PSG-sheet was shaken with 1.5 mL of methanol for its conditioning (1500 rpm, 2 min) in a multitube vortex. The conditioned sorptive device was incubated in 1.5 mL of centrifugated sample at pH 10 (placed in an HPLC vial) under continuous shaking at 1500 rpm for 45 min. After the analytes´ isolation, the PSG-sheet was introduced in 1 mL of Milli-Q water for the washing step, shaking 2 min at 1500 rpm, and dried at room temperature. For the elution, the PSG-sheet was introduced in an HPLC flat insert containing 300 µL of eluent (methanol/H_2_O 50/50 (v/v) with 1% formic acid) and shaken at 750 rpm for 10 min. Finally, the PSG-sheet was removed, and the vial was directly analyzed by direct infusion mass spectrometry (DI-MS/MS). The instrumental parameters of the analysis are described in section 3 of the ESM (Tables S1 and S2).

## Results and discussion

**Fig. 1:**
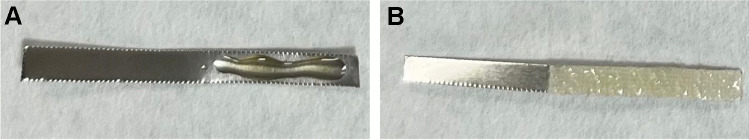
Polyester deposition on (**A**) stainless-steel substrate and (**B**) silica gel particles adhere to the substrate

The direct deposition of a stable layer of polymer on smooth substrates, such as stainless-steel, is a challenge due to the reduced wettability of its surface. Homogeneity of the coating is not easy to achieve, as the substrate is so slick that the polymer tends to concentrate in a particular area, being poorly distributed, as can be seen in Fig. 1A. The pre-immobilization of silica particles over the stainless-steel has been recently reported by our research group as a successful strategy to overcome this limitation [13]. These particles, which are immobilized using an adhesive tape, increase the superficial area of the substrate, improving also its wettability as can be observed in Fig. 1B. This strategy improves the homogeneity of the coating, also increasing the superficial area of the resulting material. Particle size is key to achieve a homogeneous But thinner polymer layer since it defines the surface area available for that coating. Different PSG-sheets were prepared using microparticles of different sizes, including 70–230 mesh (63–210 µm), 35–70 mesh (210–500 µm) and 28–200 mesh (74–600 µm). Amorphous particles were selected due to their lower price. A pre-polymer solution (further details are presented in the experimental section) was deposited onto the particulate area by drop-casting technique, and the pre-polymer was thermally cured for 30 min to achieve the final stacking of the polymer chains.

**Fig. 2:**
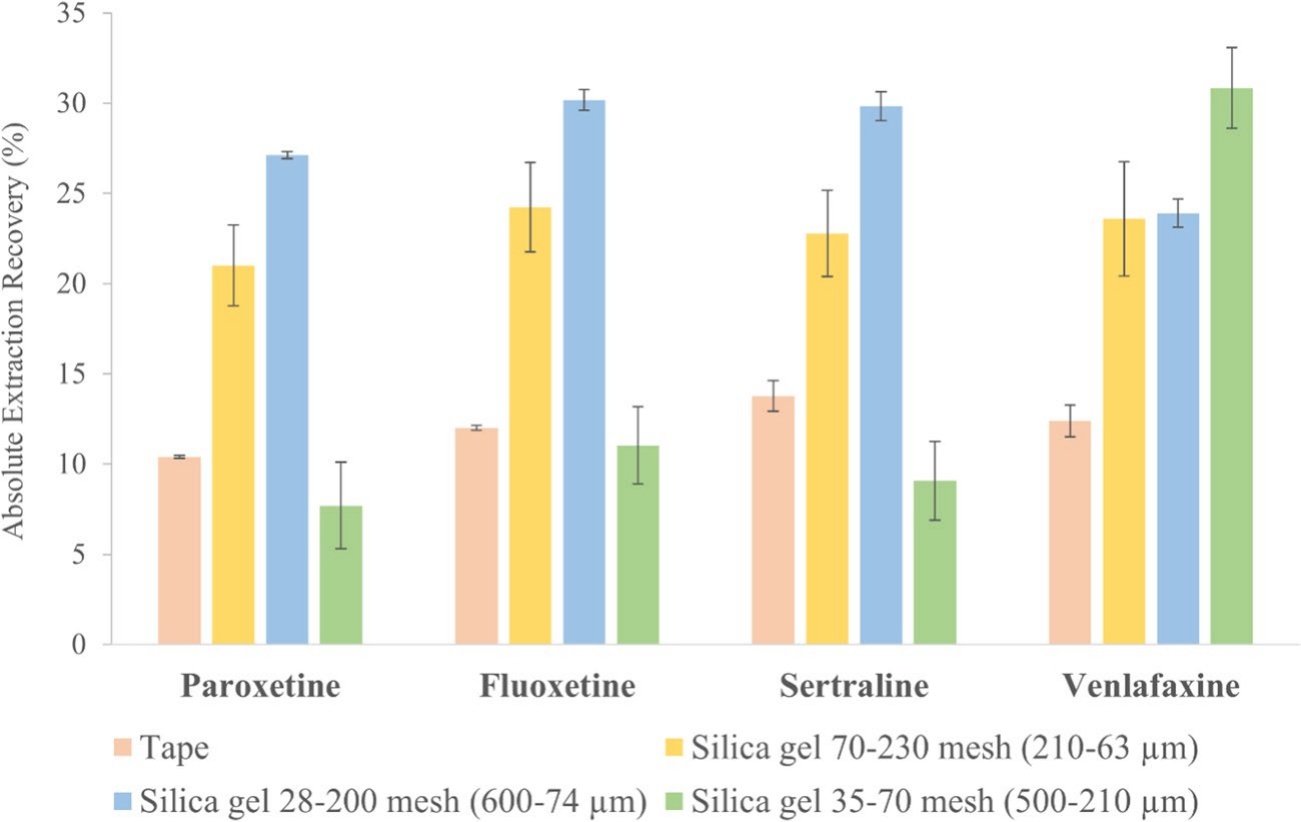
Evaluation of the particle size on the extraction efficiency of the PSG-sheets. The results were expressed as absolute extraction recovery (%) for n=3

The extraction performance of the prepared PSG-sheets was evaluated and the results, expressed as absolute extraction recovery (AER), are shown in Fig. 2. For this purpose, aqueous standard solutions containing the analytes at 50 µg·L^−1^ were incubated with the different PSG-sheets for 45 min at 750 rpm. The pH was set to 10 to promote the hydrophobic and π-π interactions between the analytes and the sorptive material. Afterwards, the isolated analytes were eluted with a methanol/water/formic acid mixture (79.5/19.5/1 v/v). For comparative purposes, the direct deposition onto the tape (without particles) were evaluated to clarify the effect of the particles on the exposed polymer area. The extraction capacity of the microparticles could not be studied since the SG-sheets (silica gel coated sheets without polymer) were not mechanically stable under agitation conditions. According to Fig. 2, the incorporation of particles enhances the extraction efficiency since they increase the surface area. Moreover, the extraction capacity was directly affected by the particle size since a fixed amount of pre-polymer was deposited onto the sheet. The particles packing degree can explain, as shows in Figure S2, the obtained results. In smallest particles, the packing degree is more compact, reducing the exposed surface area. Thus, the area provided by the particles is not sufficient to distribute 10 µL of polymer (minimum amount that can be deposited), so a thicker layer of polymer is formed, which does not sufficiently boost the particle effect. In addition, using bigger particles is also counterproductive since the polymer tends to be deposited in the voids between particles due to its viscosity, leaving a good part of them exposed to interact with the analytes. This was corroborated since venlafaxine (the most polar analyte) was mostly extracted with this particle size, being partly isolated by silica gel particles. According to the results, mixed-size particles (74–600 µm) were selected as optimal for the deposition, since by combining different sizes allows to adapt the available area to the amount of polymer deposited. Thus, larger particles provide a larger surface area, while smaller ones prevent the deposition of thick layers in the voids. Interestingly, these results showed an opposite effect to the one observed in our previous article [13], indicating that the synthesis technique (drop-casting or dip-coating), the viscosity of the polymers, and the volume of polymer deposited play a key role in the coatings.

**Fig. 3:**
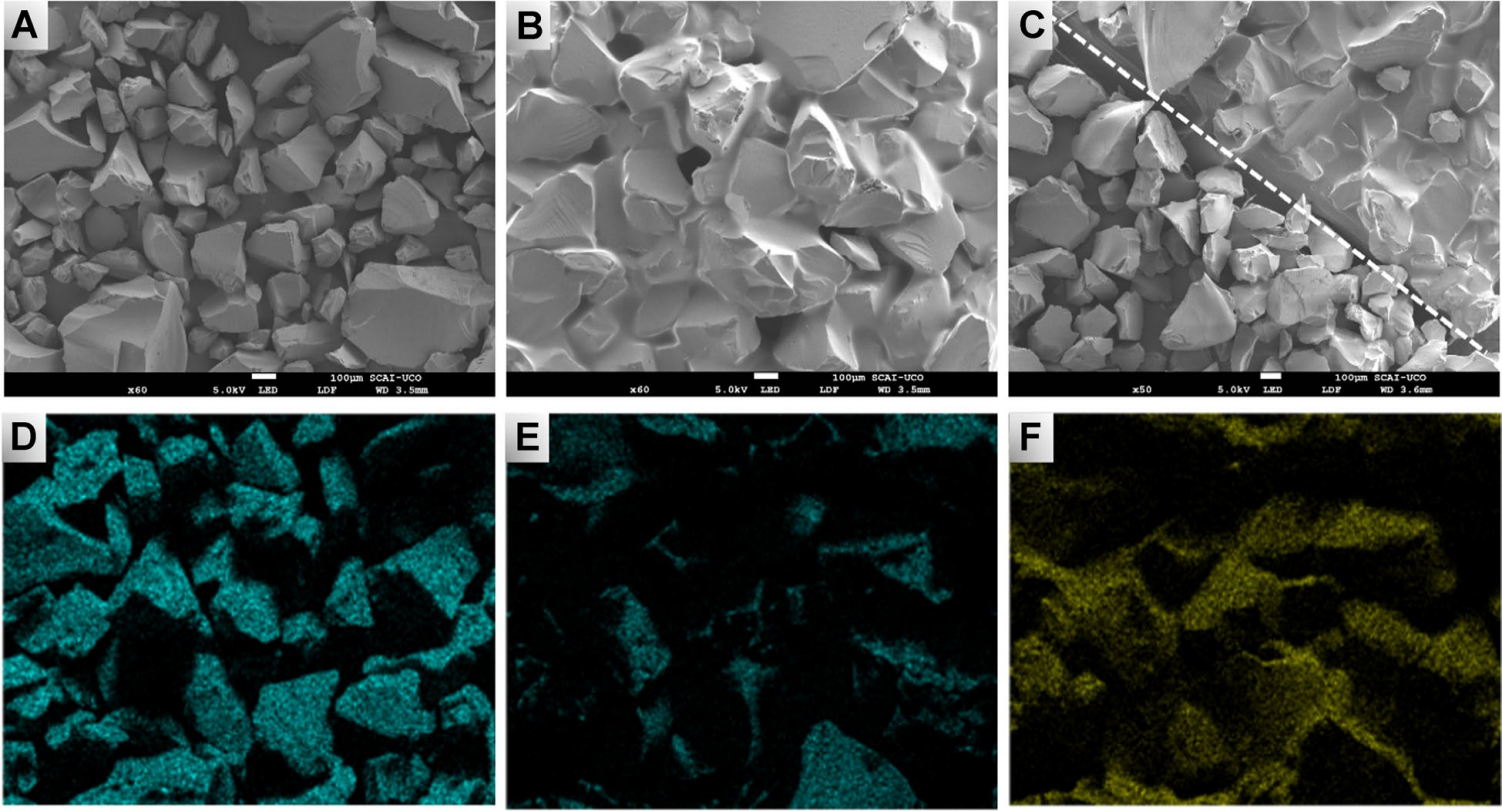
SEM micrographs of (**A**) SG-sheet and (**B**) PSG-sheet. The sheets were prepared with 28-200 mesh silica microparticles. (**C)** The difference between the cover and uncovered areas. The lower panels show the EDX analysis where the distribution of Si in (**D**) SG-sheet and (**E**) PSG-sheet is presented. (**F)** The *C* distribution in the PSG-sheet

SEM micrographs demonstrate the presence of polyester in the PSG-sheet. The particulate surface (Fig. 3A) was homogeneously coated by polymer after the deposition and curing stages (Fig. 3B). The difference is clearly observable in Fig. 3C, which shows the interface between a coated and an uncoated section.

EDX analysis of the SG- and PSG-sheets also corroborates the polymer presence. Silicon distribution is substantially higher in the raw particles (Fig. 3D) compared to the coated ones (Fig. 3E), thus demonstrating the negligible particle exposure to interact with analytes. Carbon distribution in the PSG-sheet (Fig. 3F) completes this reasoning, as it must be due to the polymeric coating.

The elution efficiency of different media was evaluated. For this, the incorporation of formic acid (1% v/v) and acidified water (20, 50 and 80 percent of water, also with formic acid at 1% v/v) in the eluent was tested, as shown in Figure S3. Although methanol can break non-polar interactions, the elution ability is negligible due to the strong retention of the analytes. The incorporation of formic acid enhances the elution by charging the amino groups of the analytes, thus weakening the analytes-material interactions. Moreover, the incorporation of a portion of acidified water in the eluent can enhance the solubilization of the charged analytes in the media, up to 50 percent of water in the elution mixture. After that, the AER decreases since the proportion of organic media is not enough to break the interactions.

### Variables related to the synthesis

Once the isolation-elution of the analytes was evaluated, the parameters directly involved in the synthesis and deposition of the polyester were studied. The synthesis of the pre-polymer can be followed by a color change from light yellow to brown, getting darker and more viscous as time elapses. ATR-IR measurements were used to continuously monitor pre-polymer synthesis. Figure S4 summarizes the ATR-IR spectra obtained from time 0 (a mixture of unreacted monomers) up to 5 h of synthesis, while a detailed discussion can be found in section 6 of ESM. For a proper deposition, the degree of polymerization should not be too high to allow easy handling of the pre-polymer. The synthesis time is a variable closely related to the synthesis course. For this, a pre-polymer with 1:10 molar ratio was synthesized, obtaining some aliquots after 1, 2, and 3 h, being consequently deposited onto the sheet and cured to remove the non-reacting diol. Longer synthesis time provides a very viscous mixture, which is not practical for a proper deposition. The extraction capacity of the obtained PSG-sheets was evaluated, and the results are presented in Figure S5. According to the results, the AER decreases over time, But the differences are not so significative, so 1 h was considered as optimal time.

**Fig. 4:**
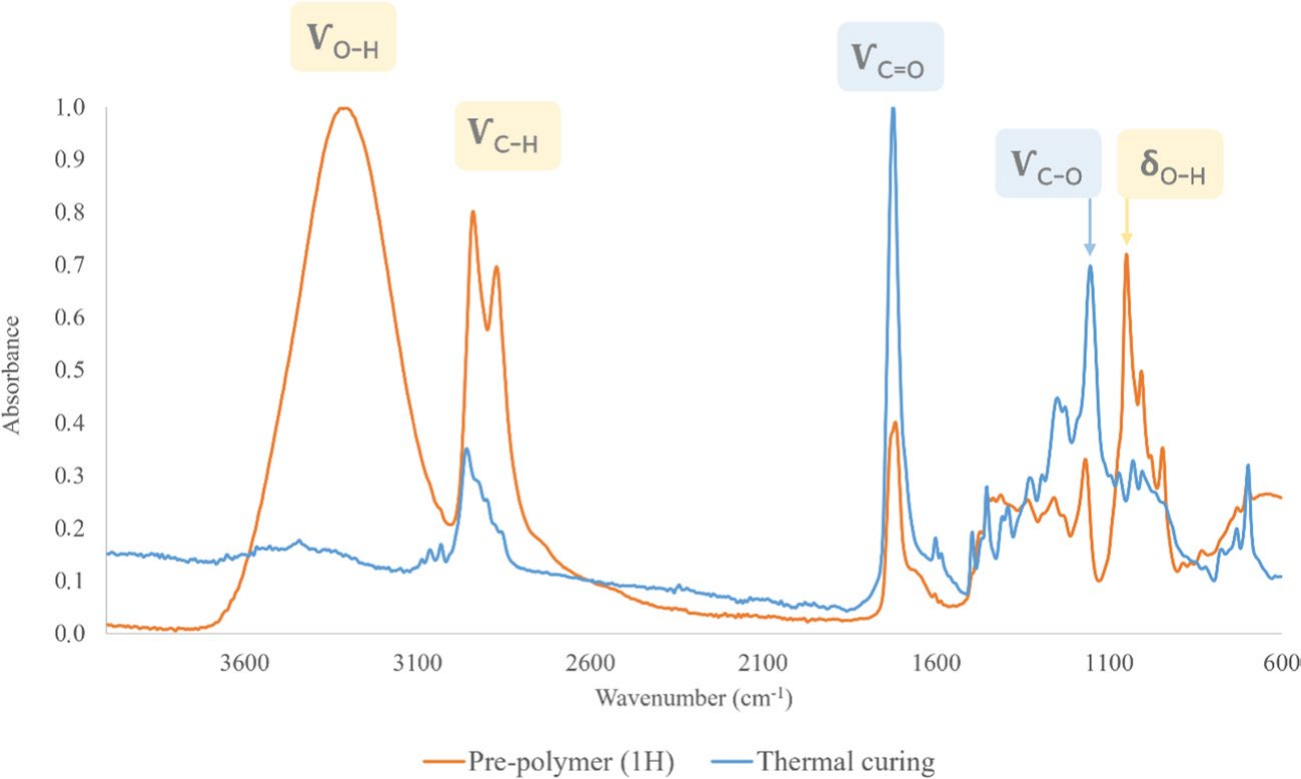
ATR-IR spectra of the polyester pre- and post- curing at 235 ºC, highlighting the characteristic bands of both monomers, 1,4-butanediol (yellow color) and phenylsuccinic acid (blue color)

The curing stage is key to achieving polymer immobilization. After the synthesis, the pre-polymer is soluble in methanol because the polyester chains are dispersed in the synthesis medium, being necessary to force the inter-chain linking. Polyester chain stacking is favored by π-π interactions between the aromatic systems of phenylsuccinic acid [15], being totally necessary for the close approximation of them for this purpose. Since the synthesis is carried out in excess of diol, it is necessary to remove the unreacted alcohol for chains approximation. Due to its high boiling temperature (230 ºC), a thermal stable tape is necessary to attach the particles to the stainless-steel sheet. Figure 4 shows the ATR-IR spectra of the polyester before and after the curing step. The more striking fact is the complete disappearance of O–H stretching after the thermal treatment, demonstrating the successful evaporation of the alcohol that remained in excess after the reaction. In addition, symmetric C-H tension disappears, leaving only the asymmetric one, which is also of lower intensity. However, the C = O and C-O stretching remain unshifted after the curing stage, also increasing their intensity. This fact can be ascribed to the high temperature, which, in conjunction with evaporation, promotes the formation of more bonds between unreacted monomers, thus increasing the intensity of these bands.

The effect of curing time on the extraction capacity of the PSG-sheet was evaluated in Figure S6. A detailed discussion of the results can be found in Section 7 of ESM.

Finally, the reproducibility of the synthesis procedure was verified in Section 8 of ESM (Figure S7).

### Variables related to the extraction procedure

Once the synthesis procedure was studied, different variables directly affecting the analytes retention were evaluated. The most important one is sample pH, since it defines the interaction mechanism with the analytes. Four different levels, from acidic to alkaline conditions, were evaluated in Figure S8. The best results were obtained at pH 10, since analytes are in neutral form (pH > pKa), enhancing π-π interactions with the aromatic rings of the polymer. According to the chemical features of the analytes, summarized in Table S3, hydrogen bond interactions are also present, justifying the good results obtained for venlafaxine (the most polar analyte). At alkaline conditions (pH 11.5), the AER decreases as certain leaching of the PSG-sheet is detected, causing ionic suppression of the analytes in the instrumental analysis. At pH lower than pKa values (pH 7 and 4), the analytes are positively charged, being the log K_O/W_ values lower than in the previous case. The π-π interactions are less favored, and the AER decreases. At neutral pH, some exposed silica gel particles could contribute to the isolation of the analytes. The surface silanol groups of the particles are negatively charged at this pH, establishing ionic interactions with the positive analytes [18]. However, the AER is higher at alkaline conditions, indicating that the interaction with the silica gel particles is not favored at the extraction conditions since the silica active sites are poorly accessible.

Ionic strength (Figure S9) and extraction kinetics (Figure S10) were also evaluated and they are discussed in detail in sections 10 and 11 of the ESM.

### Application of the extraction performance to oral fluids analysis

#### Sample dilution

Saliva samples are complex matrices with a pronounced matrix effect. The high viscosity of the matrix negatively affects the diffusion of the analytes toward the material, thus decreasing the extraction kinetics. The dilution of the sample can partially overcome this effect, but this step leads to a reduction in the concentration of the analytes, being a major problem if the sample contains the analytes at very low concentrations. The effect of the sample dilution was tested at four different levels (non-diluted, 1/2 v/v, 1/4 v/v, and 1/6 v/v), and the analytical signal of the isolated analytes was compared (Figure S11). The results show that saliva dilution reduces the concentration of the analytes, thus decreasing the analytical signal as a function of the applied dilution factor. To boost the sensitivity level, non-diluted saliva was selected, and in-matrix calibration was performed to normalize the matrix effect on the analytical performance.

#### Analytical features of the proposed method

The developed approach was evaluated following the ICH M10 validation guideline [19], and the results are summarized in Table 1. For this purpose, blank saliva samples were spiked with the target compounds and their isotopically labeled surrogates at known concentrations and tested by applying the optimized method. A 1/x weight calibration model was built for each analyte by processing nine concentration levels in triplicate. The limits of detection (LOD) and quantification (LOQ) were calculated by a signal-to-noise ratio of 3 and 10, respectively. The linear range covers the interval LOQ-600 µg·L^−1^, with R^2^ better than 0.986. Higher concentrations than 600 µg·L^−1^ were not evaluated since the analytes are typically below this level in real samples. The precision, expressed as relative standard deviation (RSD), was evaluated at four concentration levels, namely LOQ, 5 µg·L^−1^(low concentration quality control), 200 µg·L^−1^(medium concentration quality control), and 600 µg·L^−1^(high concentration quality control). The intra-day precision was evaluated by using five different replicates per concentration level, obtaining RSD better than 8.2 at LOQ and 5.1 for the other quality controls (QCs). The inter-day precision was evaluated by extracting three different replicates per concentration level in three different days, obtaining RSD better than 16.5 and 8.7 for the LOQ and QCs, respectively. These results cover the batch-to-batch variability in the PSG-sheet fabrication since the material was prepared daily basis. The intra-day accuracy, expressed as relative recovery (RR), was evaluated by analyzing five different replicates per concentration level. The RR was in the range 87–123 at LOQ and 92–114 for the QCs. Dilution integrity (Dil QC) was evaluated at a concentration four times higher than the high concentration QC, then diluting the sample 10 times with a blank matrix, ensuring that it fits inside the linear range of the method. Precision and accuracy were evaluated by analyzing 5 replicates, obtaining RSD better than 3.4 and RR in the range 94–112, fulfilling the criteria required for precision and accuracy by the validation guideline. Thus, samples that are outside the linear range can be diluted and reanalyzed.

**Table 1:** Analytical characteristics of the approach for the determination of the target analytes in saliva samples

Analyte	LOD (µg·L^−1^)	LOQ **(**µg·L^−1^**)**	**R**^**2**^	Lineal Range** (**µg·L^−1^**)**	RSD intra-day (%, *n* = 5)					RSD inter-day (%, *n* = 9)				Accuracy (% Relative Recovery,* n *= 5)				
					LOQ	LQC	MQC	HQC	Dil QC	LOQ	LQC	MQC	HQC	LOQ	LQC	MQC	HQC	Dil QC
Fluoxetine	0.6	2	0.992	2–600	8.2	1.3	1.2	0.6	1.7	7.3	8.0	2.3	8.7	87 ± 7	106 ± 2	114 ± 2	109 ± 1	94 ± 2
Paroxetine	0.6	2	0.986	2–600	6.0	3.1	1.3	2.0	2.8	10.3	2.0	6.0	6.3	123 ± 2	98 ± 3	114 ± 3	105 ± 4	112 ± 4
Sertraline	0.6	2	0.990	2–600	3.1	1.7	1.3	2.1	3.4	12.9	3.8	5.5	4.3	116 ± 4	94 ± 2	109 ± 2	104 ± 3	95 ± 4
Venlafaxine	0.6	2	0.990	2–600	4.3	5.1	1.2	2.6	1.9	16.5	3.2	3.9	5.0	110 ± 4	92 ± 5	106 ± 2	100 ± 3	94 ± 2

*LOD*, limit of detection; *LOQ*, limit of quantification; *RSD*, relative standard deviation; *LQC*, low quality control (5 µg·L^−1^**)**; *MQC*, medium quality control (200 µg·L^−1^**);** *HQC*, high quality control (600 µg·L^−1^**),** Dil QC, dilution quality control (2400 µg·L^−1^**)**

LOQ and LOD were calculated taking a signal-to-noise ratio of 3 and 10 respectively. The intra-day precision was calculated using five independent extractions at each value. The inter-day precision was calculated using three independent extractions in three different days. The same approach was used to determine the accuracy of the method

The stability of the analytes in the sample matrix was studied at two different concentrations levels (low QC and high QC). A detail discussion is presented in section 13 of ESM.

The applicability of the method was tested by analyzing a real saliva sample from a patient under continuous paroxetine treatment. The sample was acquired 3.5 h after the intake of 20 mg of paroxetine in pill format. The approach allows the correct quantification of the analyte, as shown in Table 2.

**Table 2:** Application of the approach to one sample from a volunteer under paroxetine intake

Analyte	Sex	Dosage consumed (mg)	Time since consumption (h)	Concentration found (µg·L^−1^)
Paroxetine	Male	20	3.5	7.2 ± 0.5

The deviation was expressed as expanded uncertainty for a confidence level of 95% (coverage factor K = 2) using intra-day methodological uncertainty calculated at a level close to the concentration found in the sample

The developed method was compared with others found in literature with the same target compounds (Table S5) [20–24]. The most important advantage is the improvement of the sample throughput because I) most of the methods use chromatographic separation, thus increasing the analysis time, and II) the number of samples processed per hour is generally lower due to the manual processing, with some exceptions indicated in the table. In addition, the LOD achieved is generally lower than the counterparts, except for the µ-SPE method. However, the possibility of processing up to 90 samples at the same time is advantageous for the sample throughput compared to that approach. The precision and accuracy values are also comparable to the others. It should be also noted that the linear range obtained with the proposed method is wide, encompassing concentrations from a few µg·L^−1^ to high concentrations, thus providing versatility for the real samples analysis.

#### Evaluation of the sustainability and applicability of the method

The greenness of the method was evaluated by using AGREEprep (analytical greenness for sample preparation tool) [25] because this metric is primarily focused on the sample preparation step. The results are presented in Figure S12. A final score of 0.6 was obtained, thus demonstrating the sustainability of the method. As can be seen in Figure S12, the evaluation penalized certain aspects of the method that should be the focus of future work. These aspects include ex-situ sample treatment, the use of semi-automatic systems that require operator intervention, and the use of materials that are affordable but non-renewable, such as the substrate itself. This tool was also used to evaluate the greenness of each method compared in Table S5 from a sustainability perspective. As can be seen in the table, the score obtained for the other methods is generally worse than the present approach because most of them include evaporation-reconstitution steps, which also involves high solvent consumption and time required to process the sample.

The applicability of the method was evaluated by using BAGI (blue applicability grade index tool) [26], which considers the practical aspects of White Analytical Chemistry. The results, which are shown in Figure S13, provided a good score of 60. Both metrics highlight the maximization of the sample throughput due to the possibility of processing many samples in the same batch, combined with a fast analysis by DI-MS/MS.

## Conclusions

This article evaluates the synthesis of polyester-coated metallic sheets (PSG-sheets) by drop-casting technique. The guiding substrate concept was successfully applied for this purpose using amorphous silica gel microparticles as a template. The particles were stably retained with double sided adhesive tape, allowing to increase the wettability and the surface area of the substrate without prior aggressive etching procedures. The polyester was easily synthesized by conventional free melt polycondensation using phenylsuccinic acid and 1,4-butanediol as monomers, enabling the preparation of above 100 sorptive phases with only 1 mL of polymer. The active role of the polyester in the retention of the analytes was fully demonstrated by determining four SSRI (fluoxetine, paroxetine, sertraline, and venlafaxine) in saliva samples by direct infusion mass spectrometry analysis (DI-MS/MS). The method allows the determination of the target compounds at the low µg·L^−1^ range, and it was successfully applied to analyze one saliva sample from a patient under paroxetine medical prescription. The fast analysis combined with the simultaneous extraction of up to 90 samples in the same batch results advantageous for the sample throughput.

The preparation of the PSG-sheets stands out for its affordability since it starts from readily available and inexpensive materials. In addition, the synthesis method of the polyester can be adapted to other monomers available in the laboratory, considering the nature of the analytes to be determined, thus enabling tuneable chemistry.

## Supplementary Information

Below is the link to the electronic supplementary material.

Supplementary Material 1 (DOCX 2.71MB)

## Data Availability

The authors declare that the data supporting the findings of this study are available within the paper and its Supplementary Information files. Should any raw data files be needed in another format they are available from the corresponding author upon reasonable request.

## References

[1] Kasperkiewicz A, Pawliszyn J (2021) Multiresidue pesticide quantitation in multiple fruit matrices via automated coated blade spray and liquid chromatography coupled to triple quadrupole mass spectrometry. Food Chem 339:127815. 10.1016/j.foodchem.2020.127815 32836024 10.1016/j.foodchem.2020.127815

[2] Gómez-Ríos GA, Tascon M, Reyes-Garcés N et al (2018) Rapid determination of immunosuppressive drug concentrations in whole blood by coated blade spray-tandem mass spectrometry (CBS-MS/MS). Anal Chim Acta 999:69–75. 10.1016/j.aca.2017.10.016 29254576 10.1016/j.aca.2017.10.016

[3] Azizi A, Shahhoseini F, Modir-Rousta A, Bottaro CS (2019) High throughput direct analysis of water using solvothermal headspace desorption with porous thin films. Anal Chim Acta 1087:51–61. 10.1016/j.aca.2019.08.022 31585566 10.1016/j.aca.2019.08.022

[4] Azizi A, Shahhoseini F, Bottaro CS (2022) Biological matrix compatible porous thin film for quick extraction of drugs of abuse from urine prior to liquid chromatography-mass spectrometry analysis. Talanta 241:123264. 10.1016/j.talanta.2022.123264 35144113 10.1016/j.talanta.2022.123264

[5] Tascon M, Gómez-Ríos GA, Reyes-Garcés N et al (2017) Ultra-fast quantitation of voriconazole in human plasma by coated blade spray mass spectrometry. J Pharm Biomed Anal 144:106–111. 10.1016/j.jpba.2017.03.009 28318747 10.1016/j.jpba.2017.03.009

[6] Mirnaghi FS, Chen Y, Sidisky LM, Pawliszyn J (2011) Optimization of the coating procedure for a high-throughput 96-blade solid phase microextraction system coupled with LC–MS/MS for analysis of complex samples. Anal Chem 83:6018–6025. 10.1021/ac2010185 21711040 10.1021/ac2010185

[7] Kueseng P, Pawliszyn J (2013) Carboxylated multiwalled carbon nanotubes/polydimethylsiloxane, a new coating for 96-blade solid-phase microextraction for determination of phenolic compounds in water. J Chromatogr A 1317:199–202. 10.1016/j.chroma.2013.08.038 23972463 10.1016/j.chroma.2013.08.038

[8] Hu K, Zhou W, Yang C et al (2024) Rapid screening of drugs in environmental water using metal organic framework/Ti3C2Tx composite coated blade spray-mass spectrometry. J Hazard Mater 472:134609. 10.1016/j.jhazmat.2024.134609 38759280 10.1016/j.jhazmat.2024.134609

[9] Pelit F, Erbas I, Mert Ozupek N et al (2024) A novel approach utilizing rapid thin-film microextraction method for salivary metabolomics studies in lung cancer diagnosis. Microchem J 207:112069. 10.1016/j.microc.2024.112069

[10] Yan K, Liu X, Liu J et al (2024) Octadecyl-fibrous mesoporous silica nanospheres coated 96-blade thin-film microextraction for high-throughput analysis of phthalic acid esters in food and migration from food packages. J Chromatogr A 1716:464636. 10.1016/j.chroma.2024.464636 38219624 10.1016/j.chroma.2024.464636

[11] Kirschner N, Dias AN, Budziak D et al (2017) Novel approach to high-throughput determination of endocrine disruptors using recycled diatomaceous earth as a green sorbent phase for thin-film solid-phase microextraction combined with 96-well plate system. Anal Chim Acta 996:29–37. 10.1016/j.aca.2017.09.047 29137705 10.1016/j.aca.2017.09.047

[12] do Carmo SN, Merib J, Carasek E (2019) Bract as a novel extraction phase in thin-film SPME combined with 96-well plate system for the high-throughput determination of estrogens in human urine by liquid chromatography coupled to fluorescence detection. J Chromatogr B Analyt Technol Biomed Life Sci 1118:17–24. 10.1016/j.jchromb.2019.04.037 31005770 10.1016/j.jchromb.2019.04.037

[13] Pedraza-Soto AM, Lucena R, Cárdenas S (2025) Silica microparticles as guiding substrates for the fabrication of polyamide-coated stainless-steel sheets by dip-coating technique: application to the determination of opioids in saliva samples by direct infusion mass spectrometry. Anal Chim Acta 1348:343814. 10.1016/j.aca.2025.343814 40057312 10.1016/j.aca.2025.343814

[14] Kakde D, Powell LG, Bansal KK et al (2016) Synthesis, characterization and evaluation of in vitro toxicity in hepatocytes of linear polyesters with varied aromatic and aliphatic co-monomers. J Control Release 244:214–228. 10.1016/j.jconrel.2016.08.003 27498019 10.1016/j.jconrel.2016.08.003

[15] Chen W, Wu K, Qu Z, Lu M (2019) Intrinsic high thermal conductive co-polyester based on offset π-π stacking. Eur Polym J 121:109275. 10.1016/j.eurpolymj.2019.109275

[16] Bu Y, Feng J, Sun M et al (2016) Facile and efficient poly(ethylene terephthalate) fibers-in-tube for online solid-phase microextraction towards polycyclic aromatic hydrocarbons. Anal Bioanal Chem 408:4871–4882. 10.1007/s00216-016-9567-z 27173390 10.1007/s00216-016-9567-z

[17] Cai Q, Bai T, Zhang H et al (2021) Catalyst-free synthesis of polyesters via conventional melt polycondensation. Mater Today 51:155–164. 10.1016/j.mattod.2021.07.024

[18] Samiey B, Toosi AR (2010) Adsorption of malachite green on silica gel: effects of NaCl, pH and 2-propanol. J Hazard Mater 184:739–745. 10.1016/j.jhazmat.2010.08.101 20869174 10.1016/j.jhazmat.2010.08.101

[19] Committee for Medicinal Products for Human Use ICH guideline M10 on bioanalytical method validation and study sample analysis Step5 (2022) ICH guideline M10 on bioanalytical method validation Step 5 (europa.eu) Accessed 20 July 2025

[20] Marasca C, Protti M, Mandrioli R et al (2020) Whole blood and oral fluid microsampling for the monitoring of patients under treatment with antidepressant drugs. J Pharm Biomed Anal 188:113384. 10.1016/j.jpba.2020.113384 32505892 10.1016/j.jpba.2020.113384

[21] González-Galán C, Millán-Santiago J, Martínez-Pérez-Cejuela H et al (2024) Pipette-tip microextraction using carboxymethylated wooden-based sawdust sorbent for the determination of antidepressants in oral fluid by direct infusion mass spectrometry. Microchem J 207:112002. 10.1016/j.microc.2024.112002

[22] Tartaglia A, Covone S, Rosato E et al (2022) Fabric phase sorptive extraction (FPSE) as an efficient sample preparation platform for the extraction of antidepressant drugs from biological fluids. Adv Sample Prep 3:100022. 10.1016/j.sampre.2022.100022

[23] Soares S, Rosado T, Barroso M, Gallardo E (2024) Quantification of antidepressants in oral fluid and plasma samples using microextraction by packed sorbent and analysis by gas chromatography-tandem mass spectrometry. Microchem J 204:111031. 10.1016/j.microc.2024.111031

[24] de Castro A, Concheiro M, Quintela O et al (2008) LC–MS/MS method for the determination of nine antidepressants and some of their main metabolites in oral fluid and plasma. J Pharm Biomed Anal 48:183–193. 10.1016/j.jpba.2008.05.024 18602787 10.1016/j.jpba.2008.05.024

[25] Wojnowski W, Tobiszewski M, Pena-Pereira F, Psillakis E (2022) Agreeprep – analytical greenness metric for sample preparation. TrAC Trends Anal Chem 149:116553. 10.1016/j.trac.2022.116553

[26] Manousi N, Wojnowski W, Płotka-Wasylka J, Samanidou V (2023) Blue applicability grade index (BAGI) and software: a new tool for the evaluation of method practicality. Green Chem 25:7598–7604. 10.1039/D3GC02347H

