# Supplementary material for: Polyester-coated stainless-steel sheets using silica gel microparticles as surface pre-modifiers: a novel approach to determine selective serotonin reuptake inhibitors in saliva samples by direct infusion tandem mass spectrometry
Source: Mikrochim Acta. 2025 Sep 9;192(10):647. doi: 10.1007/s00604-025-07521-2 (PMC12417288; doi:10.1007/s00604-025-07521-2)
Supplement: Supplementary file 1 — Supplementary Material 1 (DOCX 2.71MB) [file 604_2025_7521_MOESM1_ESM.docx]

Polyester-coated stainless-steel sheets using silica gel microparticles as surface pre-modifiers: a novel approach to determine selective serotonin reuptake inhibitors in saliva samples by direct infusion tandem mass spectrometry

Ana M. Pedraza-Soto, Rafael Lucena, Soledad Cárdenas*

Affordable and Sustainable Sample Preparation (AS_2_P) research group, Departamento de Química Analítica, Instituto Químico para la Energía y el Medioambiente IQUEMA, Universidad de Córdoba, Campus de Rabanales, Edificio Marie Curie, E-14071, Córdoba, Spain.

Corresponding author mail: [scardenas@uco.es](mailto:scardenas@uco.es) (Prof. Cárdenas)

**1. Stock and working standards**

A stock standard solution of each analyte was prepared in methanol (Panreac, Barcelona, Spain) at 20 mg·L^-1^ and stored in the freezer. Working solutions were prepared by diluting the stocks in methanol, Milli-Q water (Millipore Corp., Madrid, Spain), or saliva, depending on their final use. A stock standard solution containing the isotopically labeled compounds was prepared in methanol at 4 mg·L^-1^ and stored in the freezer. These compounds were added to the required working solutions and real samples.

**2. PSG-sheets fabrication**

PSG-sheets were easily prepared following the procedure shown in Figure S1. First, a segment of thermal resistant double-sided adhesive tape was placed only on one side of a stainless-steel sheet. After cutting the sheet into individual units (3 mm x 3 cm), the protective layer of the adhesive tape was removed, and the glued part of the substrate was immersed in a vial containing silica gel microparticles (28-200 mesh). To prevent undesirable particle detachment, the particles poorly adhered to the surface were removed by mechanical agitation. Each sheet contains 11 ± 1 mg of particles (data calculated from 10 independent replicates). Then, 10 µL of pre-polymer was deposited onto the particulate area by the drop-casting technique using a micropipette. The sheet was subsequently cured in the oven at 235 ºC for 30 min to evaporate the excess of diol and achieve the final stacking of the polyester chains.

PSG-sheets were washed twice with 1 mL of Milli-Q water and twice with 1 mL of methanol, shaking at 1500 rpm for 5 min. The material was finally dried at room temperature before its use. PSG-sheets were prepared daily to avoid cross-contamination due to the simple and cost-effective fabrication procedure since about 100 PSG-sheets can be prepared with a single batch of pre-polymer.

**
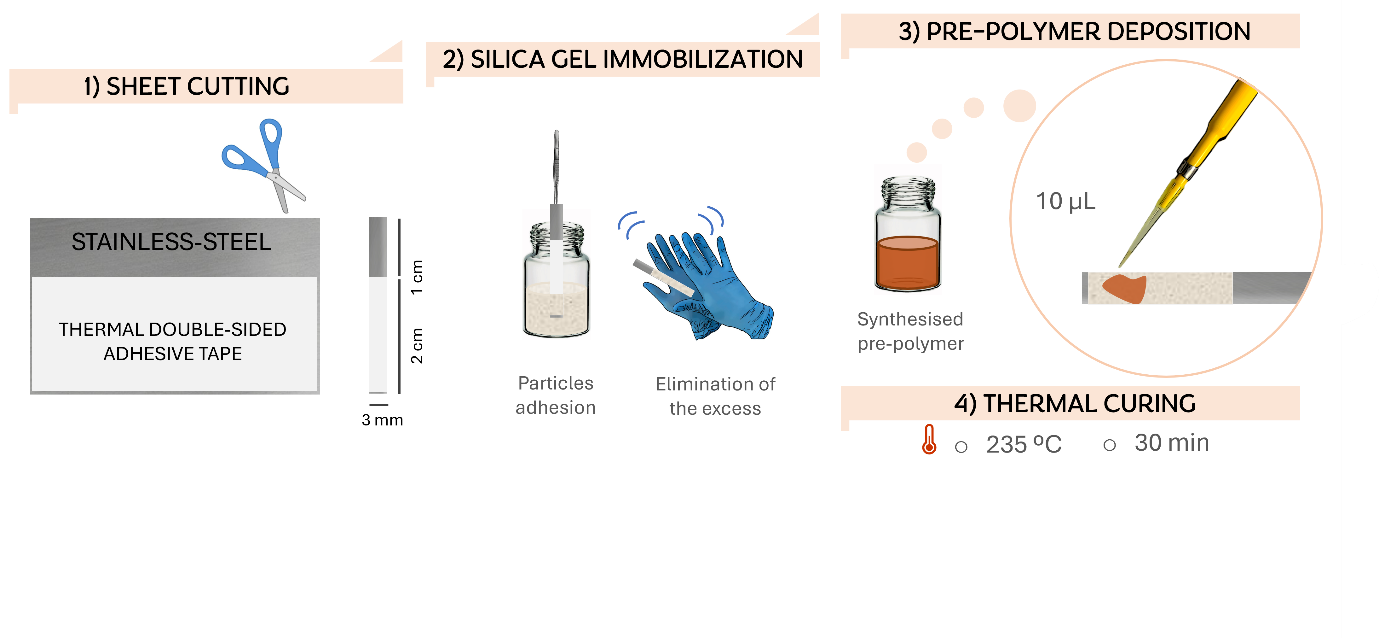
**

**Figure S1**. Scheme of the PSG-sheet fabrication.

**3**. **Mass spectrometry analyses**

The DI-MS/MS analyses were performed on an Agilent 1260 Infinity HPLC system (Agilent, Palo Alto, CA, USA). The carrier phase consists of an aqueous phase (0.1 % formic acid) and methanol in a 10:90 v/v ratio. The identification and quantification were performed in an Agilent 6420 Triple Quadrupole MS with an electrospray source. The fragmentation patterns and the instrumental parameters are shown in Table S1 and S2.

**Table S1**. Multiple reaction monitoring transitions of the target analytes.

| **Compound** | **Precursor Ion (m/z)** | **Product ion (m/z)** | **Transition** | **Fragmentor Voltage (V)** | **Collision energy (eV)** |
| --- | --- | --- | --- | --- | --- |
| **Fluoxetine** | 310.1 | 148 | Identification | 95 | 4 |
|  |  | 44.1 | Quantification |  | 10 |
| **Paroxetine** | 330.1 | 192.1 | Identification | 150 | 20 |
|  |  | 70.2 | Quantification |  | 34 |
| **Sertraline** | 306.1 | 274.9 | Identification | 95 | 9 |
|  |  | 158.9 | Quantification |  | 30 |
| **Venlafaxine** | 278.1 | 260.1 | Identification | 125 | 8 |
|  |  | 58.2 | Quantification |  | 18 |

**Table S2**. Multiple reaction monitoring transitions of the isotopically labeled compounds.

| **Compound** | **Precursor Ion (m/z)** | **Product ion (m/z)** | **Transition** | **Fragmentor Voltage (V)** | **Collision energy (eV)** |
| --- | --- | --- | --- | --- | --- |
| **Fluoxetine-d6** | 316.1 | 154 | Identification | 95 | 5 |
|  |  | 44.2 | Quantification |  | 10 |
| **Paroxetine-d6** | 336.1 | 198.1 | Identification | 85 | 20 |
|  |  | 76.2 | Quantification |  | 34 |
| **Sertraline-d3** | 309.1 | 275 | Identification | 65 | 9 |
|  |  | 159 | Quantification |  | 30 |
| **Venlafaxine-d6** | 284.2 | 64.2 | Quantification | 115 | 18 |

**4. Polymer deposition details**

**
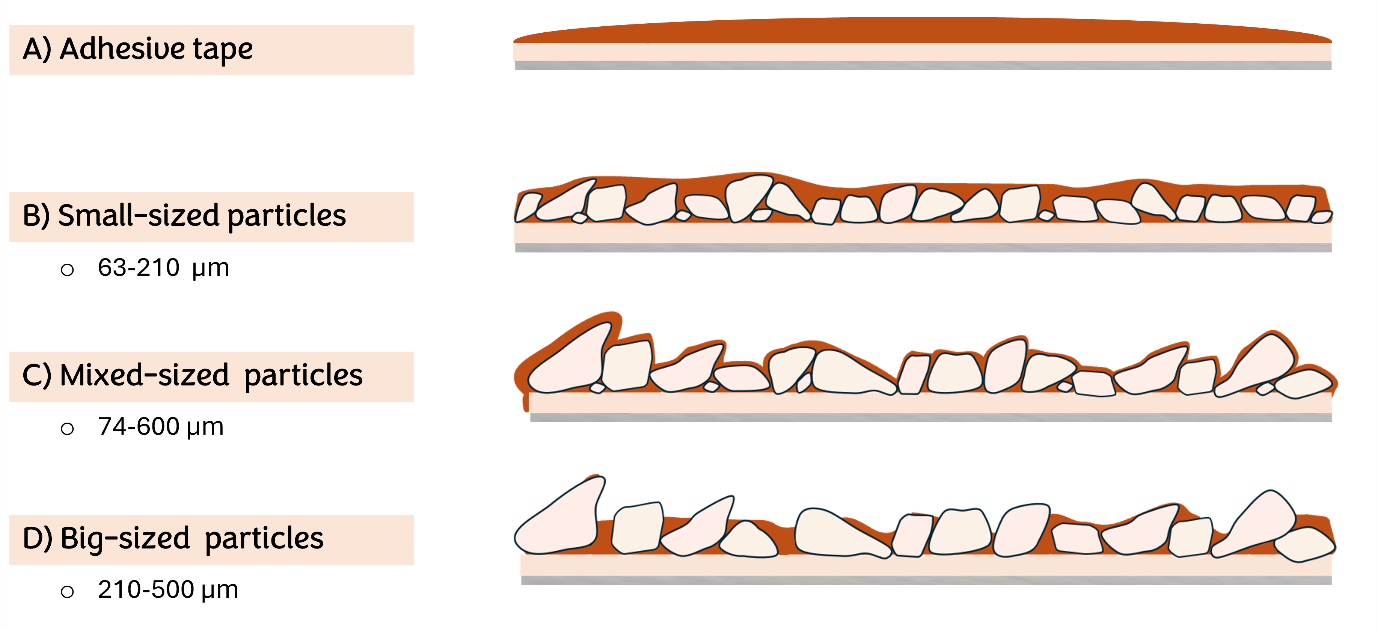
**

**Figure S2**. Effect of the guiding particle size in the polymeric layer formed after the deposition.

**5**. **Eluent selection**


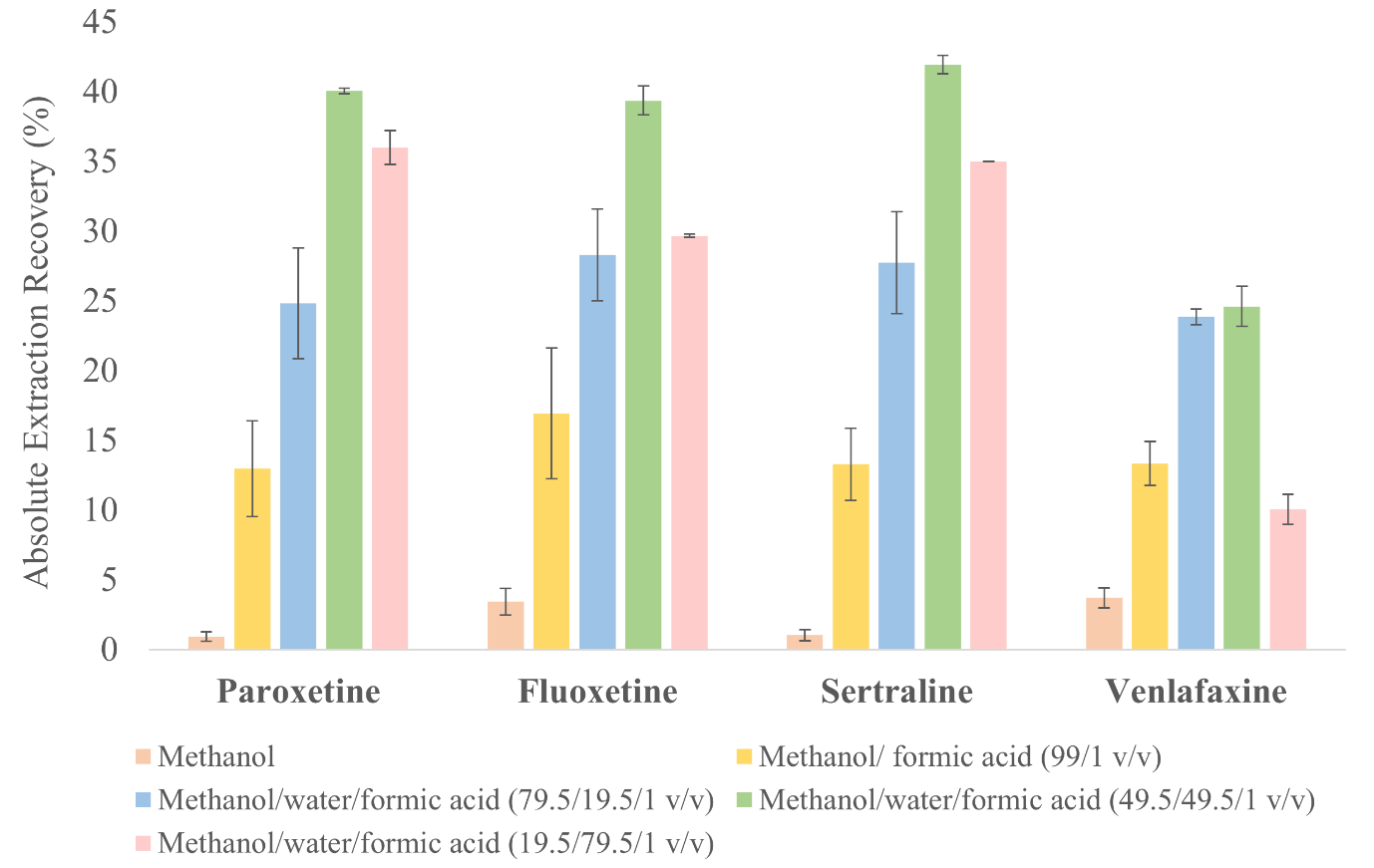


**Figure S3**. Elution ability of methanol, methanol/formic acid (99/1) and methanol/acidified water at different proportions. The results were expressed as absolute extraction recovery (%) for n=3.

**6**. **Evaluation of the pre-polymer synthesis**

As described in the main text, the polymerization degree plays a key role in the proper polymer deposition. The pre-polymer synthesis was continuously monitored by ATR-IR measurements. Figure S4 summarizes the ATR-IR spectra obtained from time 0 (mixture of unreacted monomers) up to 5 hours of synthesis. As can be seen in panel A of the figure, at the starting point (0H), the spectra present characteristic bands from both the diol and diacid. On the one hand, O-H stretching (3296 cm^-1^), asymmetric (2937 cm^-1^) and symmetric (2870 cm^-1^) C-H stretching, and in-plane O-H bending (around 1000 cm^-1^) are bands characteristic of the diol. In addition, C=O (1709 cm^-1^) and C-O (1174 cm^-1^) stretching belong to the diacid. After 1 hour of synthesis, a shift of the diacid bands suggested the successful covalent union between the monomers. In particular, the C=O stretching band is displaced to a higher wavenumber, indicating that the carbonyl groups belong to an ester. This band is wider than at time 0, probably due to the unreacted diacid remaining in the mixture. The substitution of the hydrogen in the carboxyl group (O=C-OH) by the aliphatic chain of the diol (O=C-OR) increases the negative electron density due to the introduction of the hydrocarbon chain R with electrodonor inductive effect (+I), thus reducing the distance between C=O and shifting the C=O stretching band to higher wavenumbers. In addition, the C-O stretching is also displaced, but to lower wavenumbers, because in this case, the O-R bond would be longer than the O-H bond due to the size of the group.

Panel B of the figure compares different synthesis times to show the polymerization degree in the mixture. As can be seen, times between 1 and 3 hours do not cause changes in the diol characteristic bands, but the progressive increase of the intensity in the C=O and C-O stretching bands indicates that the degree of polymerization increases with time. After 5 hours, the intensity of these bands has increased substantially, indicating that more bonds have been formed. In addition, the C=O stretching band is narrower, indicating that the remaining diacid is currently negligible. In this case, the intensity of the diol bands significantly decreases, indicating that secondary reactions of dehydration and cyclization of the diol occur to a large extent due to prolonged exposure time to the high temperature of the synthesis. This fact is responsible for the dark brown color of the pre-polymer, and the increase in viscosity, which makes it very difficult to handle the pre-polymer and its deposition onto the SG-sheet.

**
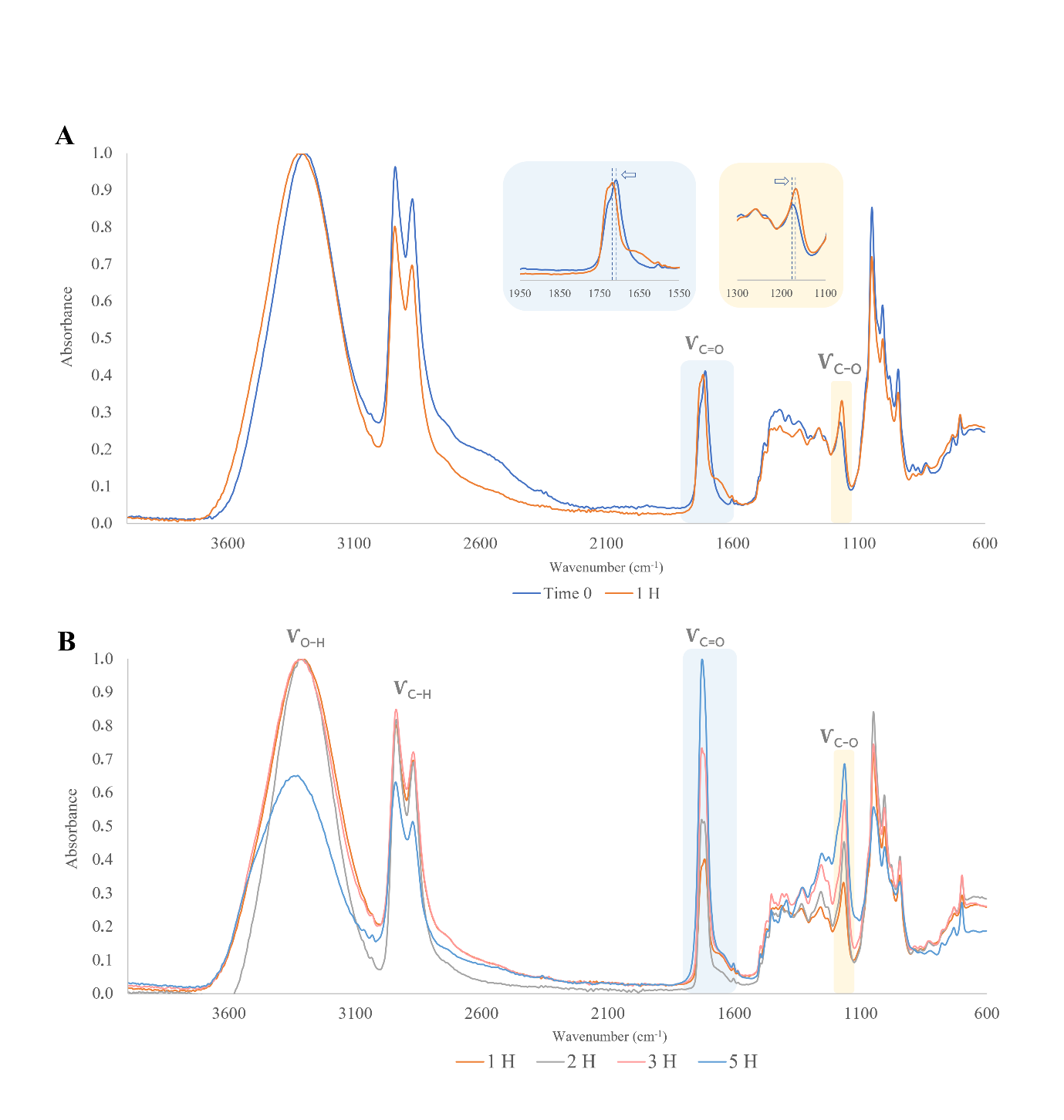
Figure S4**. ATR-IR spectra of the pre-polymer obtained (A) in the first hour of reaction and (B) after 1,2,3, and 5 hours of synthesis.


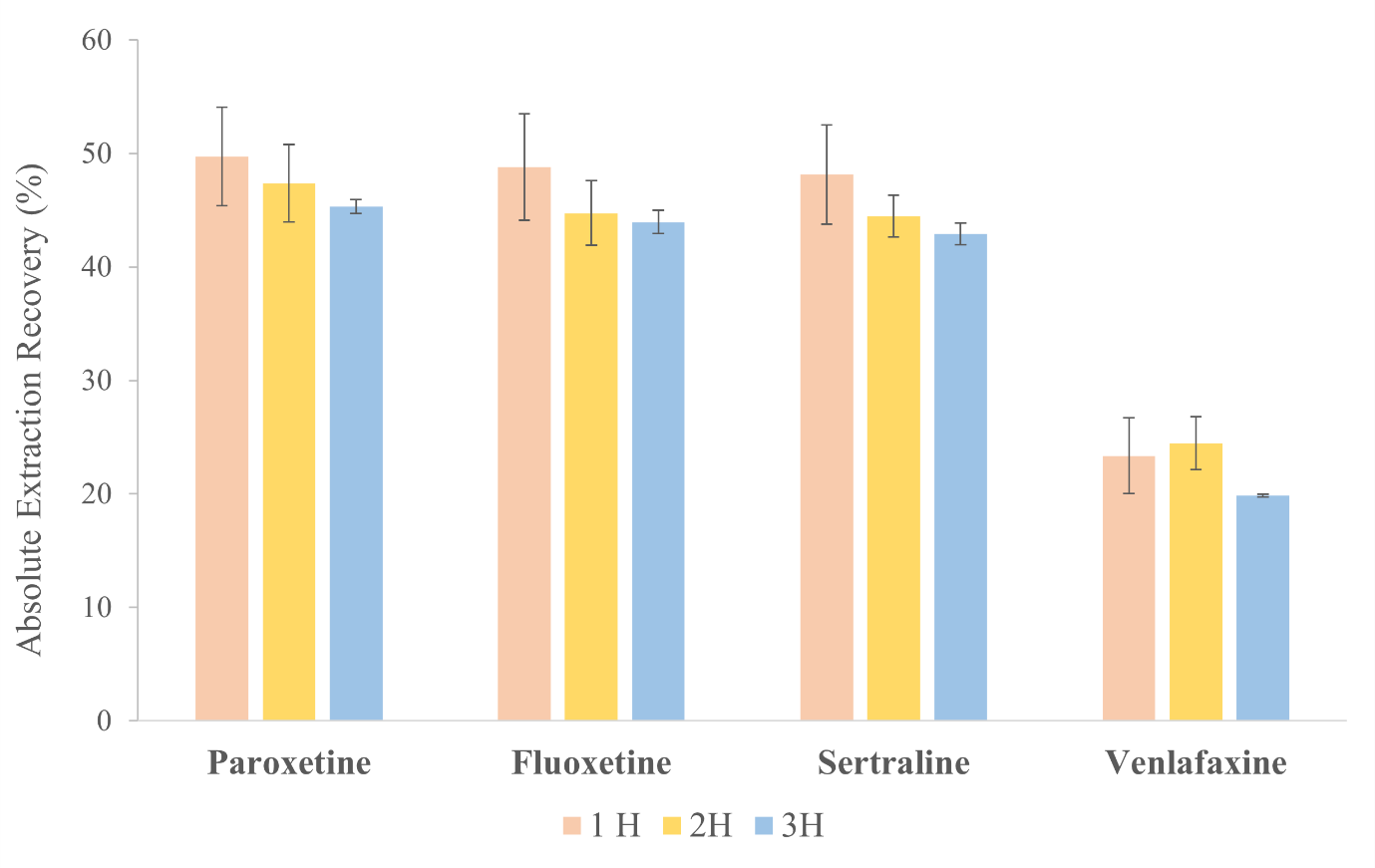


**Figure S5**. Effect of time in the pre-polymer synthesis with 1:10 diacid:diol molar ratio on the extraction efficiency of the PSG-sheets. The results were expressed as absolute extraction recovery (%) for n=3.

**7**. **Evaluation of curing time**

The effect of curing time on the extraction capacity of the PSG-sheet was evaluated in Figure S6. Although there were no significant differences between the ATR-IR spectra of polymers obtained by varying the curing time, a clear effect on the AER can be seen. According to the results, 5 min is not enough time to evaporate all the solvent, so part of pre-polymer was solved in the post-synthesis washes. After that, the AER increases with time up to 60 min, except for venlafaxine, which reaches the maximum at 15 min. For this, 30 min was selected as the optimal time to consider both trends.


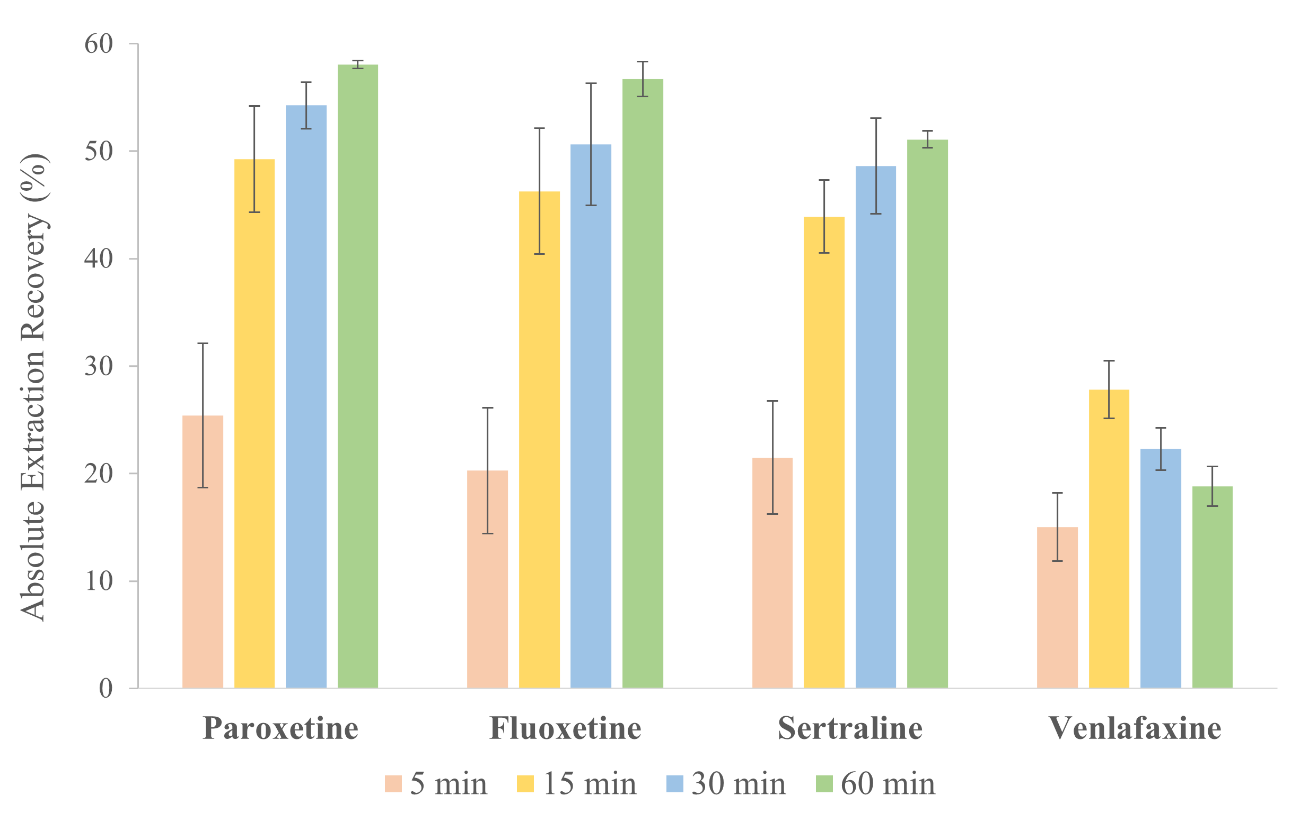


**Figure S6**. Effect of curing time on the extraction efficiency of the PSG-sheets. The results were expressed as absolute extraction recovery (%) for n=3.

**8**. **Batch-to-batch variability**

The reproducibility of the synthesis procedure was evaluated by studying the batch-to-batch variability. For this, three different pre-polymers were synthesized and deposited onto the SG-sheets, and the extraction capacity was evaluated (Figure S7). The results shown in Figure S7-A ensure the reproducibility of the synthesis since there are no significative differences between the three batches studied. Moreover, these minor differences can be normalized by using an internal standard in the extraction, as can be seen in Figure S7-B.

**
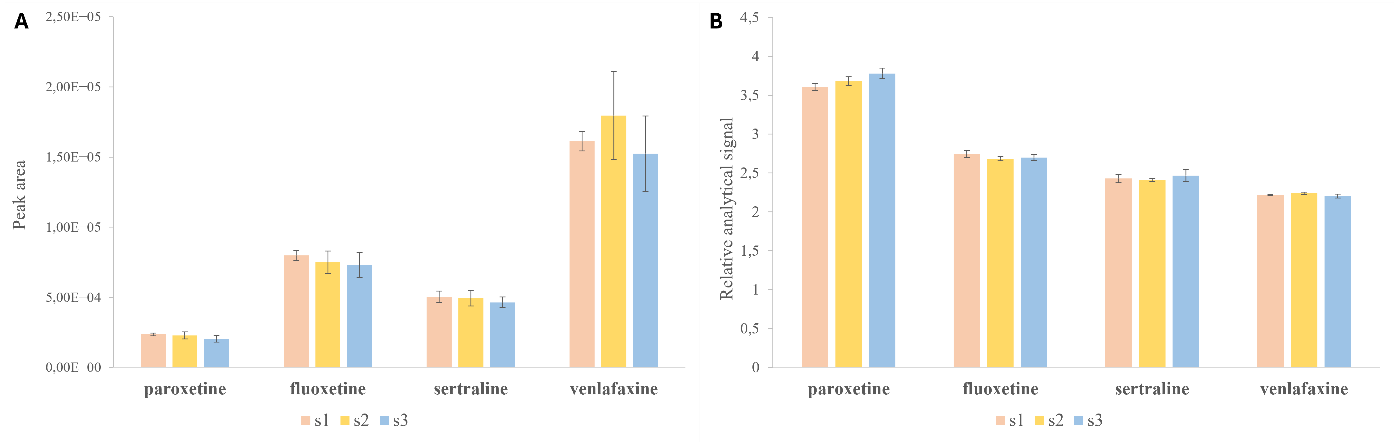
**

**Figure S7**. Evaluation of the reproducibility of the pre-polymer synthesis on the extraction efficiency of the PSG-sheet expressed as (A) peak area and (B) relative analytical signal (signal of the analyte divided by the signal of the internal standard). The results were obtained for n=3.

**9**. **pH study**

**Table S3**. Chemical characteristics of the analytes that justify the extraction trends.

| **Analyte** | **pKa** | **Log (K_O/W_)**  **_(pH 10)_** | **Hydrogen Acceptor Count** | **Hydrogen Donor Count** |
| --- | --- | --- | --- | --- |
| Fluoxetine | 9.80 | 4.6 | 2 | 1 |
| Paroxetine | 9.77 | 3.6 | 4 | 1 |
| Sertraline | 9.85 | 5.1 | 1 | 1 |
| Venlafaxine | 8.91 | 2.7 | 3 | 1 |


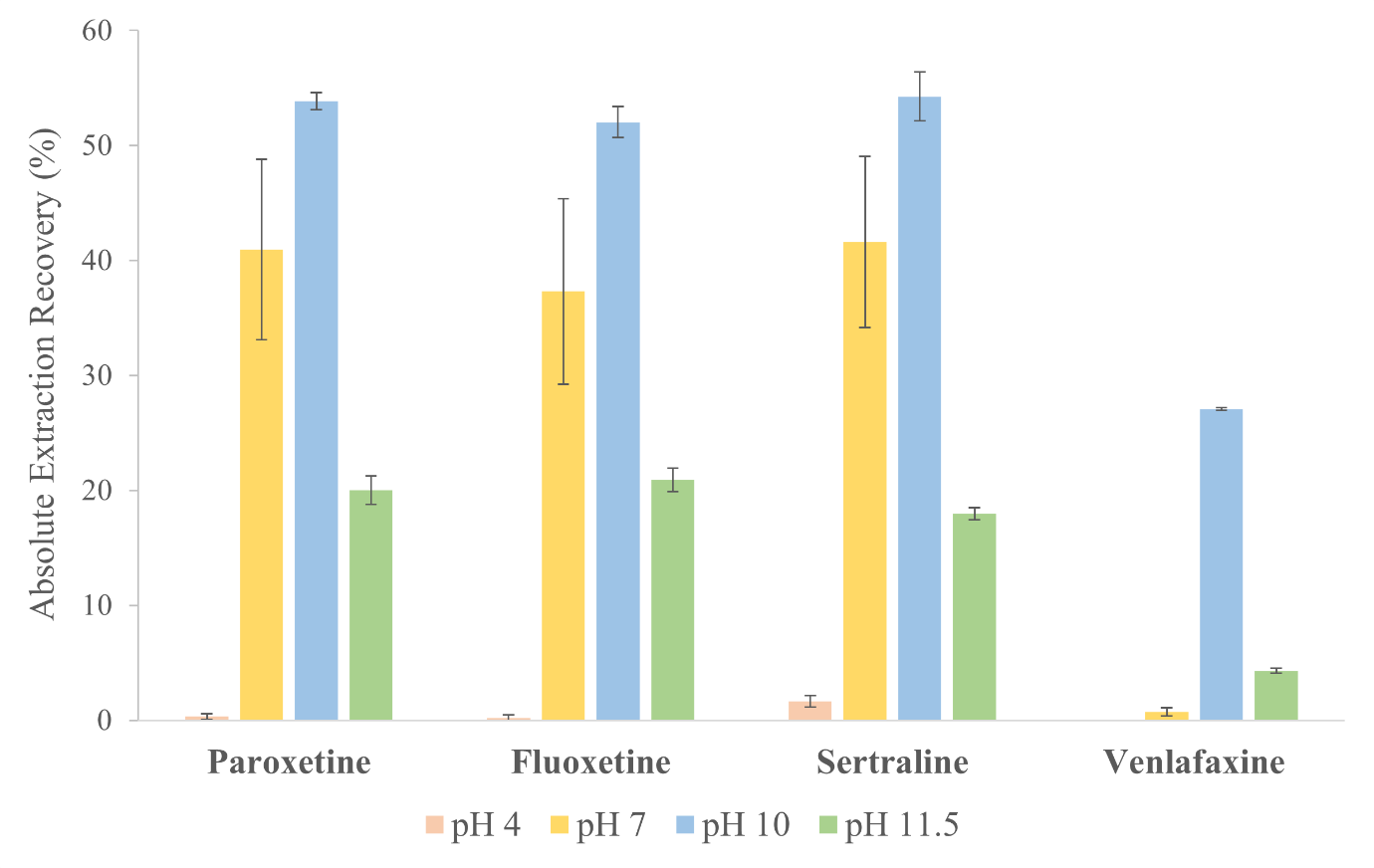


**Figure S8**. Effect of sample pH on the extraction efficiency of the PSG-sheets. The results were expressed as absolute extraction recovery (%) for n=3.

**10**. **Ionic Strength evaluation**

The ionic strength was evaluated by extracting aqueous standards of the analytes containing different NaCl concentrations as model electrolyte. As can be seen in Figure S9, ionic strength negatively affects the retention of the analytes, although the effect is not very pronounced in concentrations lower than 0.10%, venlafaxine excepted. However, the introduction of an internal standard can correct this effect. Although not all the percentages have exactly the same relative signal, the decrease respect the 0% NaCl value does not exceed 7% for all the analytes. To overcome this fact, in matrix-calibration is proposed, thus considering matrix effects.

**
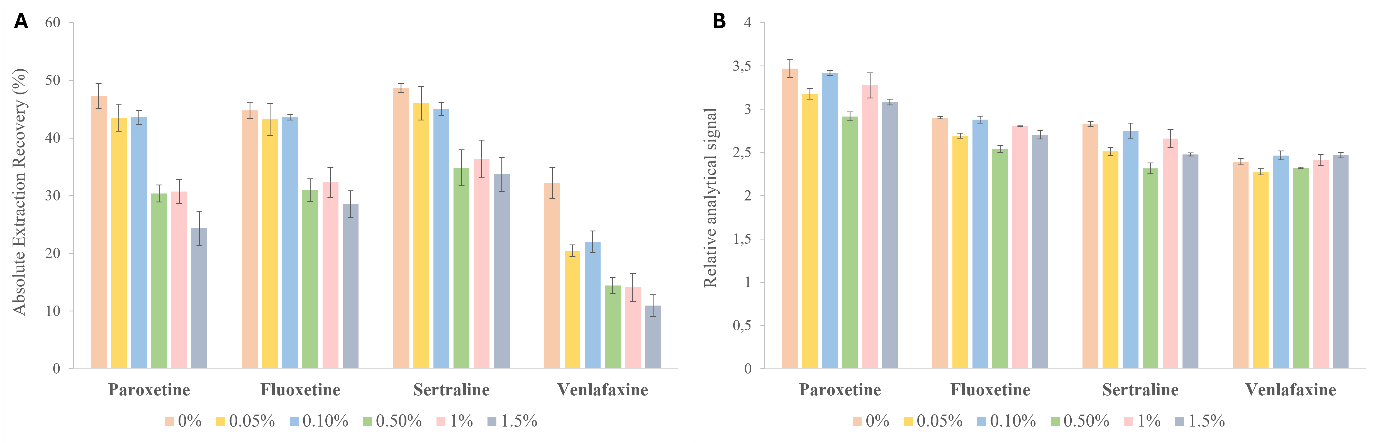
**

**Figure S9**. Effect of the ionic strength on (A) the absolute extraction recovery of the analytes and (B) the relative analytical signal (signal of the analyte divided by the signal of the internal standard). The extractions were carried out from aqueous standard solution by triplicate.

**11**. **Extraction kinetics evaluation**

The extraction kinetics was considered by combining the extraction time and the agitation speed, both with a direct influence on the analytes’ retention. Different times were evaluated at two agitation speeds (750 and 1500 rpm). As can be seen in Figure S10, at 750 rpm the AER increases over time until 45 min, when the extraction equilibrium is achieved, and then it is reversed. Increasing the speed to 1500 rpm, 10 min is sufficient to achieve the same AER, then increasing with time until 45 min. Thus, 45 min and 1500 rpm were selected as optimal parameters to improve the extraction efficiency. Although the extraction time is quite high, the possibility of extracting up to 90 samples at the same time ensures an effective sample throughput.

**
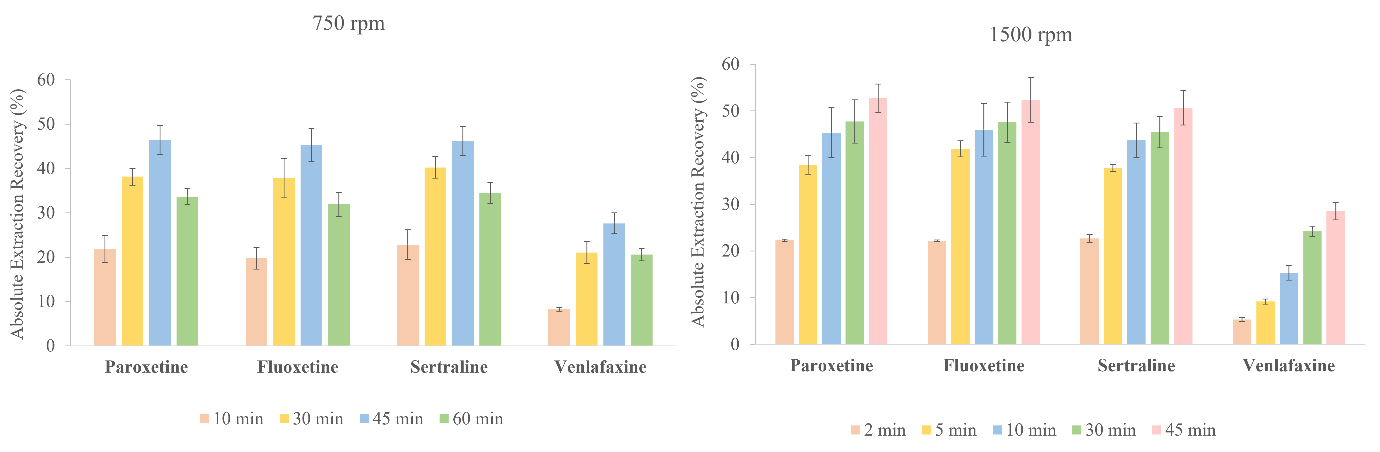
**

**Figure S10**. Evaluation of the extraction kinetics on the extraction efficiency of the analytes at different times, shaking at 750 and 1500 rpm. The results were expressed as AER (%) by triplicate.

**12**. **Saliva dilution effect in the analytical signal**

**
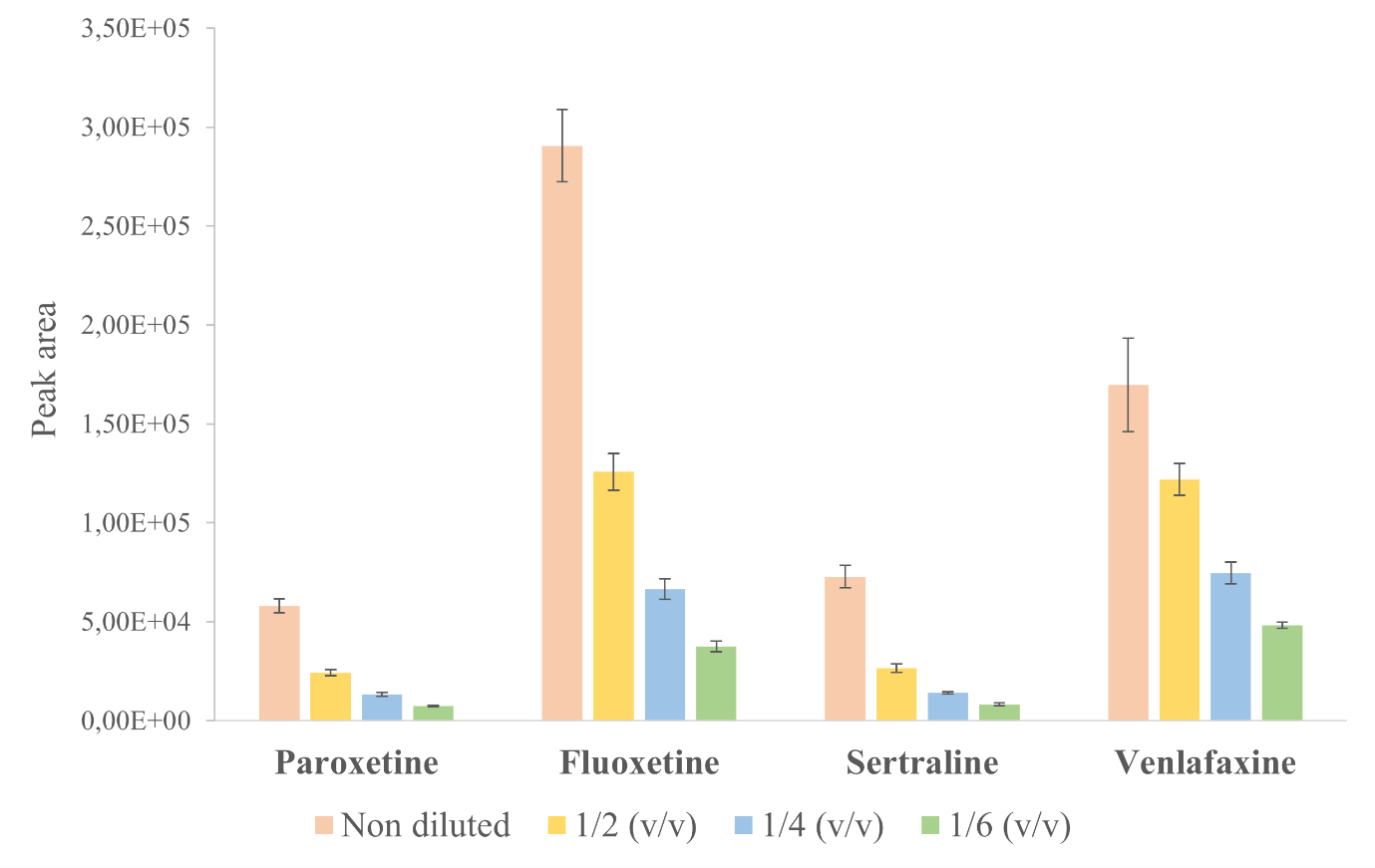
**

**Figure S11.** Effect of the sample dilution on the peak area of the analytes. A blank saliva samples spiked with the analytes at 50 µg·L^-1^ was used in these studies. Each dilution factor was studied in triplicate.

**13**. **Evaluation of the analytes´ stability in the sample matrix**

The stability of the analytes in the sample matrix was studied at two different concentrations levels (low QC and high QC). The samples were prepared and stored under different temperature conditions (room temperature, 4ºC and -20ºC), being analysed for three consecutive days in triplicate, evaluating the accuracy after each day. To evaluate the accuracy at -20ºC, three freeze-thaw cycles were performed. For this, the samples were prepared and frozen for at least 12 hours. After that, all the samples were thaw, and the accuracy were evaluated in triplicate (firs cycle). The remaining samples were refrozen, and the procedure was repeated for the second cycle. The samples were fully analysed in a third and final cycle. The results are summarized in Table S4. As can be seen, the stability depends on the concentration level. When the analytes are present in the sample at low concentration (Low QC), paroxetine and venlafaxine were stable in all storage conditions for 72 hours. The variations observed may be due to the independent addition of IS on the day of analysis in addition to the inter-day variation of the method itself. Fluoxetine and sertraline were not stable from the beginning at room temperature and in the refrigerator. In the freezer, these analytes are no longer stable after the first freezing cycle, indicating that samples containing these analytes should be kept in the freezer and fully analysed after the first thawing. For the high QC, the results were more favourable. All the analytes were stable in all the conditions, sertraline excepted. The stability of this analyte can be ensured only 24 hours at room temperature, 48 hours in the fridge, and can be subjected to two frozen-thaw cycles.

**Table S4**. Study of the stability of the retained analytes in different conditions expressed as Relative Recovery (%).

| **Temperature** | **Cycle** | **Analyte** | **5 µg·L^.1^** | **600 µg·L^.1^** |
| --- | --- | --- | --- | --- |
| Room temperature | 1 | Fluoxetine | 64 ± 4* | 93 ± 3 |
|  |  | Paroxetine | 114 ± 6 | 99 ± 5 |
|  |  | Sertraline | 75 ± 6* | 84 ± 1 |
|  |  | Venlafaxine | 101 ± 3 | 91 ± 1 |
|  | 2 | Fluoxetine | 48 ± 7* | 86 ± 2 |
|  |  | Paroxetine | 97 ± 9 | 95 ± 2 |
|  |  | Sertraline | 42 ± 11* | 77 ± 3* |
|  |  | Venlafaxine | 89 ± 7 | 89 ± 3 |
|  | 3 | Fluoxetine | 51 ± 10* | 83 ± 3 |
|  |  | Paroxetine | 92 ± 8 | 86 ± 2 |
|  |  | Sertraline | 52 ± 5* | 70 ± 1* |
|  |  | Venlafaxine | 93 ± 13 | 86 ± 1 |
| 4 ºC | 1 | Fluoxetine | 60 ± 3* | 89 ± 3 |
|  |  | Paroxetine | 99 ± 2 | 98 ± 1 |
|  |  | Sertraline | 73 ± 4* | 84 ± 3 |
|  |  | Venlafaxine | 94 ± 4 | 85.8 ± 0.6 |
|  | 2 | Fluoxetine | 55 ± 2* | 93 ± 3 |
|  |  | Paroxetine | 108 ± 4 | 98 ± 2 |
|  |  | Sertraline | 64 ± 3* | 85.5 ± 0.1 |
|  |  | Venlafaxine | 85 ± 10 | 89 ± 2 |
|  | 3 | Fluoxetine | 55 ± 1* | 85 ± 4 |
|  |  | Paroxetine | 98 ± 8 | 93 ± 2 |
|  |  | Sertraline | 64 ± 3* | 82 ± 3* |
|  |  | Venlafaxine | 90 ± 7 | 86 ± 2 |
| -20 ºC | 1 | Fluoxetine | 106 ± 5 | 98 ± 1 |
|  |  | Paroxetine | 104 ± 3 | 104 ± 3 |
|  |  | Sertraline | 102 ± 3 | 95 ± 4 |
|  |  | Venlafaxine | 97 ± 2 | 94 ± 2 |
|  | 2 | Fluoxetine | 58.8 ± 0.5* | 103 ± 2 |
|  |  | Paroxetine | 104 ± 4 | 111 ± 6 |
|  |  | Sertraline | 64 ± 5* | 101 ± 2 |
|  |  | Venlafaxine | 91 ± 6 | 96.5 ± 0.5 |
|  | 3 | Fluoxetine | 49 ± 2* | 87 ± 2 |
|  |  | Paroxetine | 93 ± 4 | 90 ± 1 |
|  |  | Sertraline | 54 ± 2* | 81.0 ± 0.5* |
|  |  | Venlafaxine | 86 ± 7 | 87 ± 2 |

*****Values not fulfilling the validation criteria for accuracy.

**14**. **Comparison of the approach with other counterparts found in literature**

**Table S5**. Comparison of the approach with other methods reported in literature.

| **Analytes** | **Matrix** | **Pre-treatment** | **Instrumental technique** | **Extractant** | **Linear range**  **(µg·L^-1^)** | **LOD**  **(µg·L^-1^)** | **RSD intra-day (%)** | **RSD inter-day (%)** | **Accuracy (% RR)** | **Samples treated per hour** | **Analysis time per sample (min)** | **AGREEprep score** | **Ref.** |
| --- | --- | --- | --- | --- | --- | --- | --- | --- | --- | --- | --- | --- | --- |
| Fluoxetine  Sertraline | Saliva | MEPS | HPLC-UV-FL | C2 sorbent | 7-750  5-500 | 2.5  1.5 | < 7.5  < 7.9 | < 8.4  < 7.9 | 91-96  90-95 | 0.3 | 17 | 0.44 | [20] |
| Fluoxetine | Saliva | µ-SPE-PT | DI-MS/MS | SWD-CX | 0.6-320 | 0.2 | < 9.1 | 3.8 | 91-102 | 20 | 1.5 | 0.56 | [21] |
| Fluoxetine  Paroxetine  Sertraline  Venlafaxine | Saliva | FPSE | HPLC-PDA | CW 20M | 100-20000  100-20000  100-20000  200-2000 | 40  40  40  60 | < 15 | - | 87.5-112 | 2 | - | 0.49 | [22] |
| Fluoxetine  Paroxetine  Sertraline  Venlafaxine | Saliva | MEPS | GC-MS/MS | M1 cartridges | 100-500  10-100  40-250  100-500 | 20  40  20  20 | < 6.0  < 12.2  < 8.2  < 13.1 | < 6.7  < 11.4  < 8.7  < 9.4 | 88-105  91-114  90-105  98-109 | 0.4 | 31 | 0.40 | [23] |
| Fluoxetine  Paroxetine  Sertraline  Venlafaxine | Saliva | SPE | LC-MS/MS | MCX cartridge | - | 2 | < 6.5  < 6.0  < 13.8  < 6.8 | < 14.9  < 6.8  < 10.4  < 10.0 | 96-111  98-111  98-111  101-109 | 80  (Automatic system) | 8 | 0.66 | [24] |
| Fluoxetine  Paroxetine  Sertraline  Venlafaxine | Saliva | TFME | DI-MS/MS | Polyester | 2-600 | 0.6 | < 8.2  < 6.0  < 3.4  < 5.1 | < 8.7  < 10.3  < 12.9  < 16.5 | 87-114  98-123  94-116  92-110 | 68  (Multiposition system) | 1.5 | 0.60 | This work |

LOD, limit of detection; RSD, relative standard deviation; RR, relative recovery.

**Pre-treatment**: MEPS, microextraction by packed sorbent; µ-SPE-PT, µ-solid-phase extraction pipette tip; FPSE, Fabric Phase Sorptive Extraction; MEPS, microextraction by packed sorbent; SPE, sorptive phase extraction; TFME, thin-film microextraction. **Instrumental technique**: HPLC-UV-FL, liquid chromatography with sequential spectrophotometric and spectrofluorimetric detection; DI-MS/MS, direct infusion mass spectrometry, HPLC-PDA, high performance liquid chromatography-photodiode array detection; GC-MS/MS, gas chromatography coupled to tandem mass spectrometry; LC-MS/MS, liquid chromatography tandem mass spectrometry. **Extractant:** SWD-CX, sawdust modified via carboxymethylation reaction; CW 20M, sol-gel carbowax sorbent coated on cellulose; M1 cartridges, 80% C_8_ and 20% SCX (strong cationic exchange particles); MCX cartridge, OASIS mixed mode cationic exchange cartridge.

**15**. **Evaluation of sustainability by analytical greenness metric for sample preparation tool (AGREEprep**

**
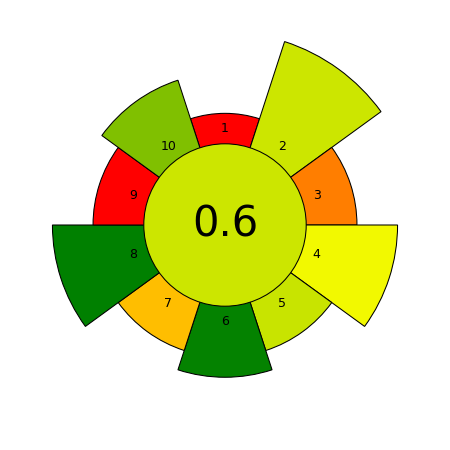
**

**
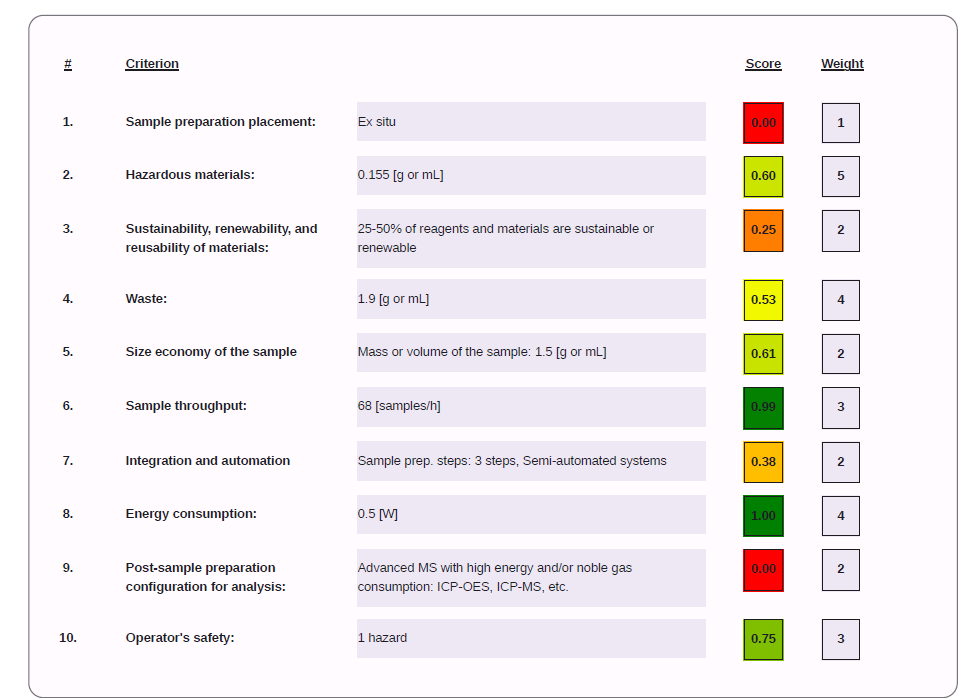
**

**Figure S12.** Evaluation of the sustainability of the proposed method using AGREEprep (analytical greenness metric for sample preparation).

**
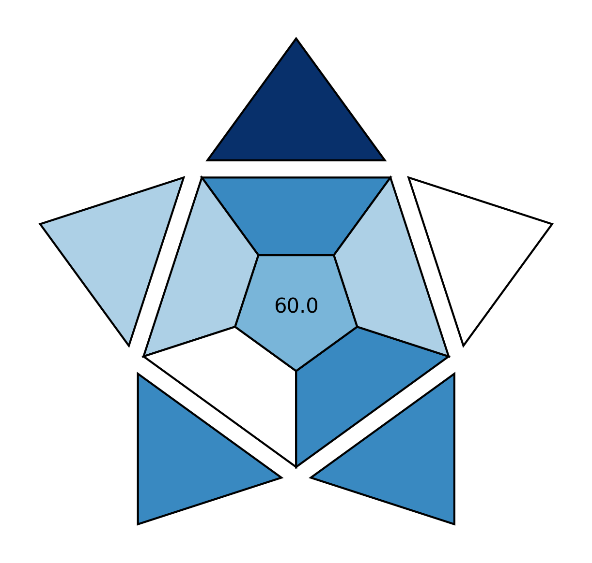
16. Evaluation of the practicality of the method by Blue applicability grade index tool (BAGI)**

**
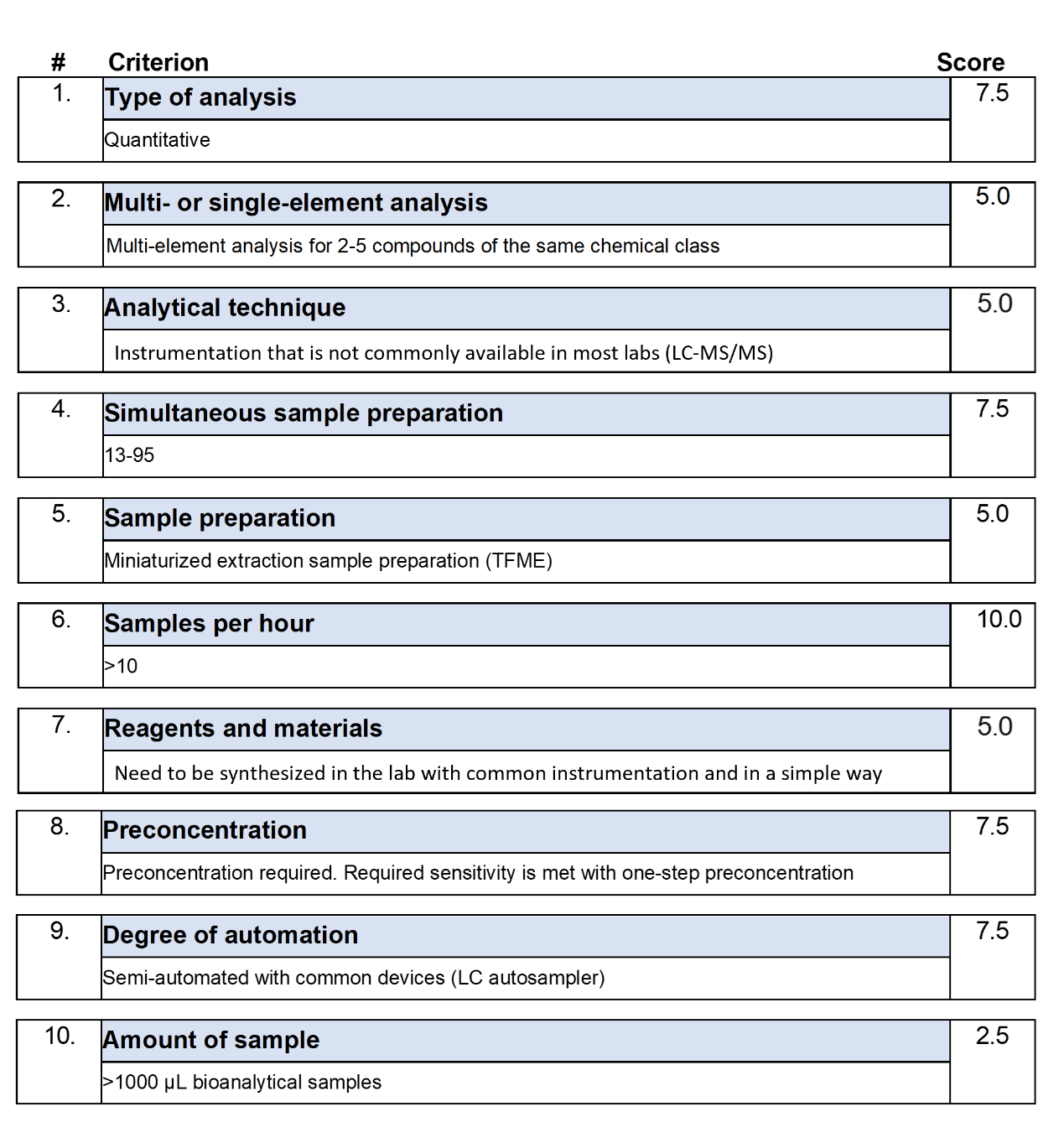
**

**Figure S13.** Evaluation of the applicability of the proposed method using BAGI (Blue applicability grade index tool).
